# A Sydnonimine‐based Click‐and‐Release Approach to Cyclic Products

**DOI:** 10.1002/chem.202500860

**Published:** 2025-04-21

**Authors:** Guillaume Force, Minghao Feng, Davide Audisio, Pierre Thuéry, Emmanuelle Schulz, Frédéric Taran

**Affiliations:** ^1^ CEA, INRAE Département Médicaments et Technologies pour la Santé (DMTS) SCBM Université Paris Saclay Gif‐sur‐Yvette 91191 France; ^2^ CEA, CNRS, NIMBE Université Paris‐Saclay Gif‐sur‐Yvette 91191 France; ^3^ Institut de Chimie Moléculaire et des Matériaux d'Orsay CNRS Université Paris‐Saclay Orsay 91400 France

**Keywords:** click‐and‐release, copper, cycloaddition, macrocycles, sydnonimines

## Abstract

Herein, we report a general, efficient, and practical methodology for the macrocyclization of compounds based on the combination of a Cu‐catalyzed click‐and‐release reaction and a hydrophobic tag. The procedure allows the preparation of cyclic products of different sizes and with various functionalities without the need of purification.

## Graphical abstract

Macrocyclick‐and‐release: The combination of a chemoselective Cu‐catalyzed click‐and‐release reaction and pyrene tags allows efficient synthesis of macrocycles and easy isolation by simple filtration through graphitic material.

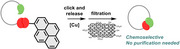

**Abbreviations**
BPDS: Bathophenanthroline disulfonate
CuAAC: Cu‐catalyzed Azide‐Alkyne Cycloaddition
NCL: Native Chemical Ligation
rGO: reduced Graphene Oxide
SPAAC: Strained‐Promoted Azide‐Alkyne Cycloaddition
TEC: Thiol‐Ene Click

## 1 Introduction

Macrocycles possess several key features that make them interesting in medical chemistry. Indeed, cyclization provides a degree of structural preorganization that favors affinity and specificity to receptors^[^ ^1^ ^]^ while providing increasing metabolic stability, membrane permeability,^[^ ^2^ ^]^ and bioavailability.^[^ ^3^ ^]^ A significant number of macrocyclic drugs are currently on the market, many are cyclic peptides^[^ ^4^ ^]^ or other complex natural products.^[^ ^5^ ^]^ Consequently, extensive efforts have been conducted to developing synthetic approaches to macrocycles using either solution‐phase chemoselective cyclization reactions^[^ ^6^ ^]^ or solid‐phase synthesis, particularly for macrocyclic peptides.^[^ ^7^ ^]^ To date many cyclic peptides are thus synthetically accessible and amenable to lead optimization via traditional medicinal chemistry efforts. However, synthetic access to many other macrocycles remains difficult and the generation of libraries through robust processes is still a challenge even if impressive examples of successful preparation of macrocycles screening collections have to be noticed.^[^ ^8^ ^]^

**Figure 1:**
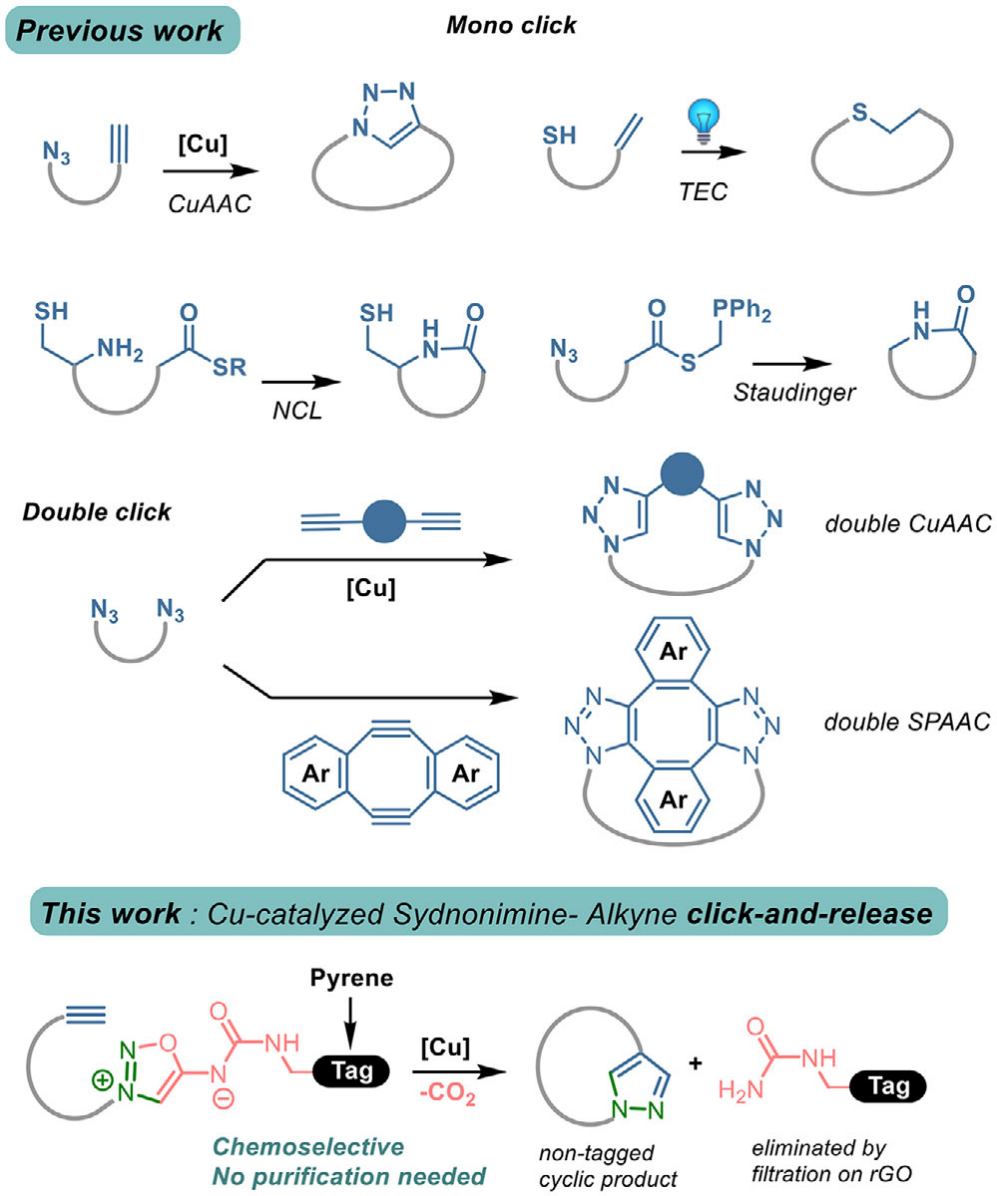
Click strategies to macrocyclization. CuAAC: Cu‐catalyzed Azide‐Alkyne Cycloaddition; SPAAC: Strained‐Promoted Azide‐Alkyne Cycloaddition; TEC: Thiol‐Ene Click; NCL: Native Chemical Ligation; rGO: reduced Graphene Oxide.

An interesting approach for improving macrocycle synthesis relies on the use of click chemistry. Due to its exceptional efficiency and selectivity, click chemistry is theoretically compatible with almost any kind of structures, making it an ideal tool for constructing complex macrocycles under mild conditions. Among the click strategies to facilitate rapid and efficient macrocyclization, the use of Cu‐catalyzed Azide‐Alkyne Cycloaddition (CuAAC),^[^ ^9^ ^]^ Thiol‐Ene click (TEC),^[^ ^10^ ^]^ Native Chemical Ligation (NCL),^[^ ^11^ ^]^ or traceless Staudinger ligation^[^ ^12^ ^]^ has been extensively employed for the cyclization of linear precursors (Figure 1). The use of double Cu‐catalyzed^[^ ^13^ ^]^ or strained promoted^[^ ^14^ ^]^ click reactions has been also described for the preparation of stapled peptides. This approach produced a range of different cyclopeptides by reacting linear diazido peptides with terminal or strained diynes stapling linkers. However, a practical bottleneck of these strategies is the need of purification after cyclization to remove residual reagents and catalyst.

In this work, we aimed to explore a click‐and‐release approach to streamline macrocycle synthesis, minimizing purification steps while maintaining high yields. Our team previously developed a click‐and‐release reaction involving sydnonimines and cyclooctynes^[^ ^15^ ^]^ and applied it in various chemical biology contexts.^[^ ^16^ ^]^ Recently, we showed that sydnonimines can also undergo click‐and‐release with terminal alkynes under Cu‐catalysis, leading to the formation of a 1, 4‐disubstitued pyrazole click product together with a released isocyanate which in turn is hydrolyzed to the corresponding amine.^[^ ^17^ ^]^ We hypothesized that the intramolecular version of this Cu‐catalyzed reaction could be particularly well‐suited for macrocyclization. The click process would enable the formation of a pyrazole‐containing macrocycle, while the simultaneous release of a tag molecule could simplify purification. Based on previous studies,^[^ ^18^ ^]^ we selected pyrene as the tagging moiety due to its chemical inertness and its ability to be efficiently removed via adsorption through π‐stacking interaction on reduced graphene oxide (rGO). Thus, compounds featuring a terminal alkyne and a sydnonimine derivatized with pyrene should undergo an intramolecular Cu‐catalyzed click‐and‐release reaction, yielding a nontagged cyclic product (Figure 1). Treating the crude reaction mixture with rGO would eliminate the released pyrene and noncyclized substrates, while an aqueous workup and extraction would remove the Cu catalyst. This approach is expected to afford the pure macrocycles without requiring labor‐intensive purification steps.

## 2 Results and Discussion

### 2.1 Development of the Method

**Figure 2:**
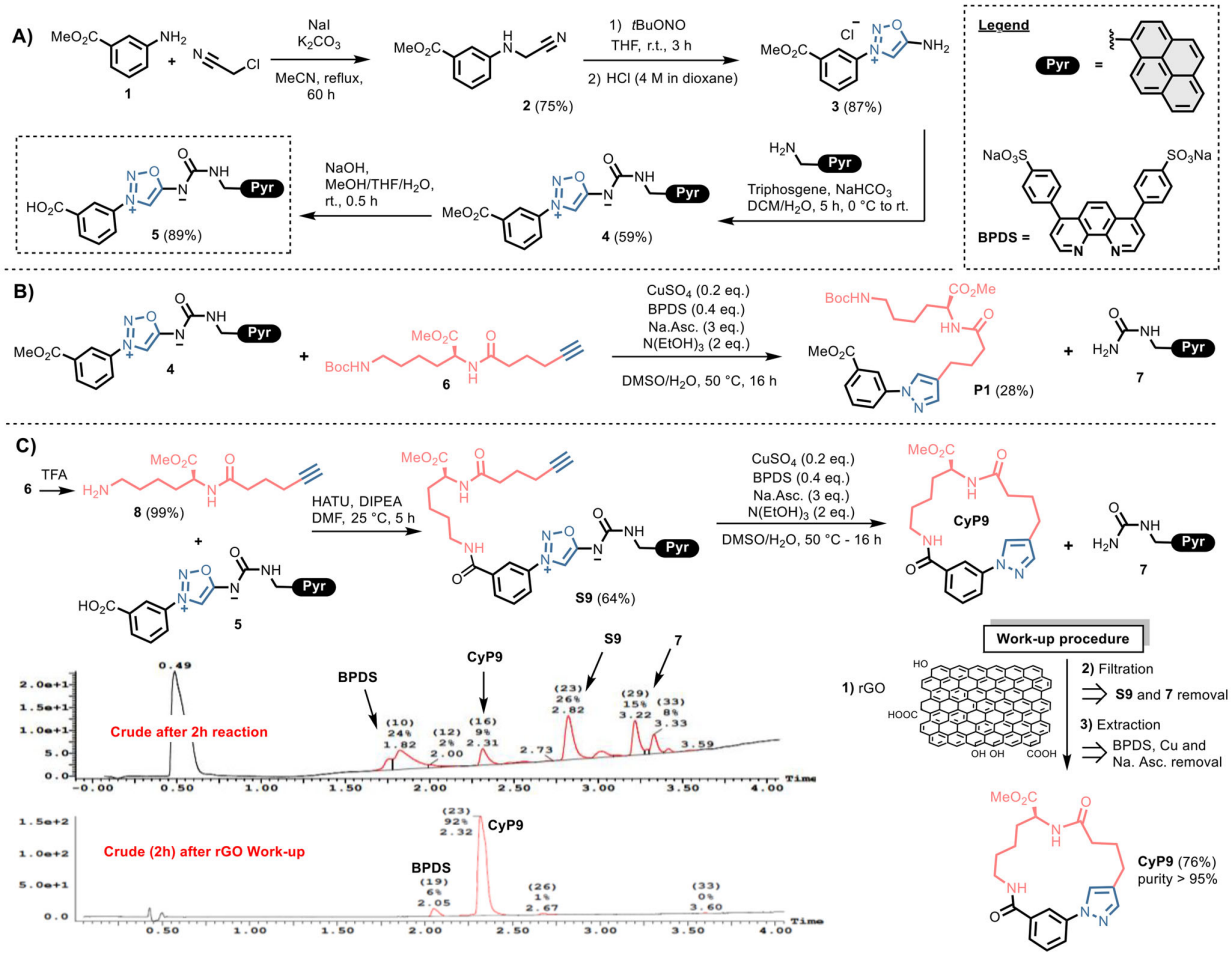
Click‐and‐release strategy to cyclic products. A) Synthesis of pyrene‐containing sydnonimine **5**. B) Intermolecular click‐and‐release reaction of sydnonimine **4** with model alkyne **6**. C) Intramolecular click‐and‐release reaction of compound **S9**, purification procedure of cyclic pyrazole products. LC/MS profiles refer to reaction crude analysis after 2 hours before (top) and after (bottom) rGO work‐up. BPDS: bathophenanthroline disulfonate.

Compound **5**, identified as the key building block for the preparation of linear precursors, was first prepared in five steps using methodologies previously described for sydnonimine preparation.^[^ ^19^ ^]^ The robustness of the synthetic route allowed an easy scale‐up to several grams of **5** (Figure 2A). Compound **5** was designed with a carboxylic function to enable attachment of alkynylated amines, leading to linear precursors ready to be cyclized.

To test our strategy, we first prepared the model linear substrate **S9** via standard peptide coupling of **8** with **5** and subjected it to copper catalysis. Pleasingly, crude mixture analysis revealed almost complete conversion of **S9** into the expected cyclic pyrazole **CyP9** after overnight reaction (Figure 2C). No dimer or other linear byproducts were detected, likely due to higher kinetics for the intramolecular reaction over the intermolecular pathway. In comparison, a control experiment in which the intermolecular reaction of compounds **4** and **6** yielded only low amounts of pyrazole **P1** (Figure 2B). We then implemented our tag‐based strategy to streamline purification of crude mixtures. LC‐MS analysis of an aliquot taken 2 hours after the start of the **CyP9** cyclization showed incomplete conversion, with detectable amounts of starting material **S9**, released urea **7** and expected **CyP9** product (Figure 2C). Adding of rGO to this aliquot successfully immobilized the pyrene‐containing compounds **S9** and **7** via π‐stacking interaction, enabling the isolation of **CyP9** as an almost pure compound (containing only traces of the ligand BPDS) after simple filtration. Applying this treatment to the crude overnight reaction mixture, followed by aqueous work‐up and extraction, provided **CyP9** in 76% isolated yield with over 95% purity without additional purification.

### 2.2 Scope of the Procedure

**Figure 3:**
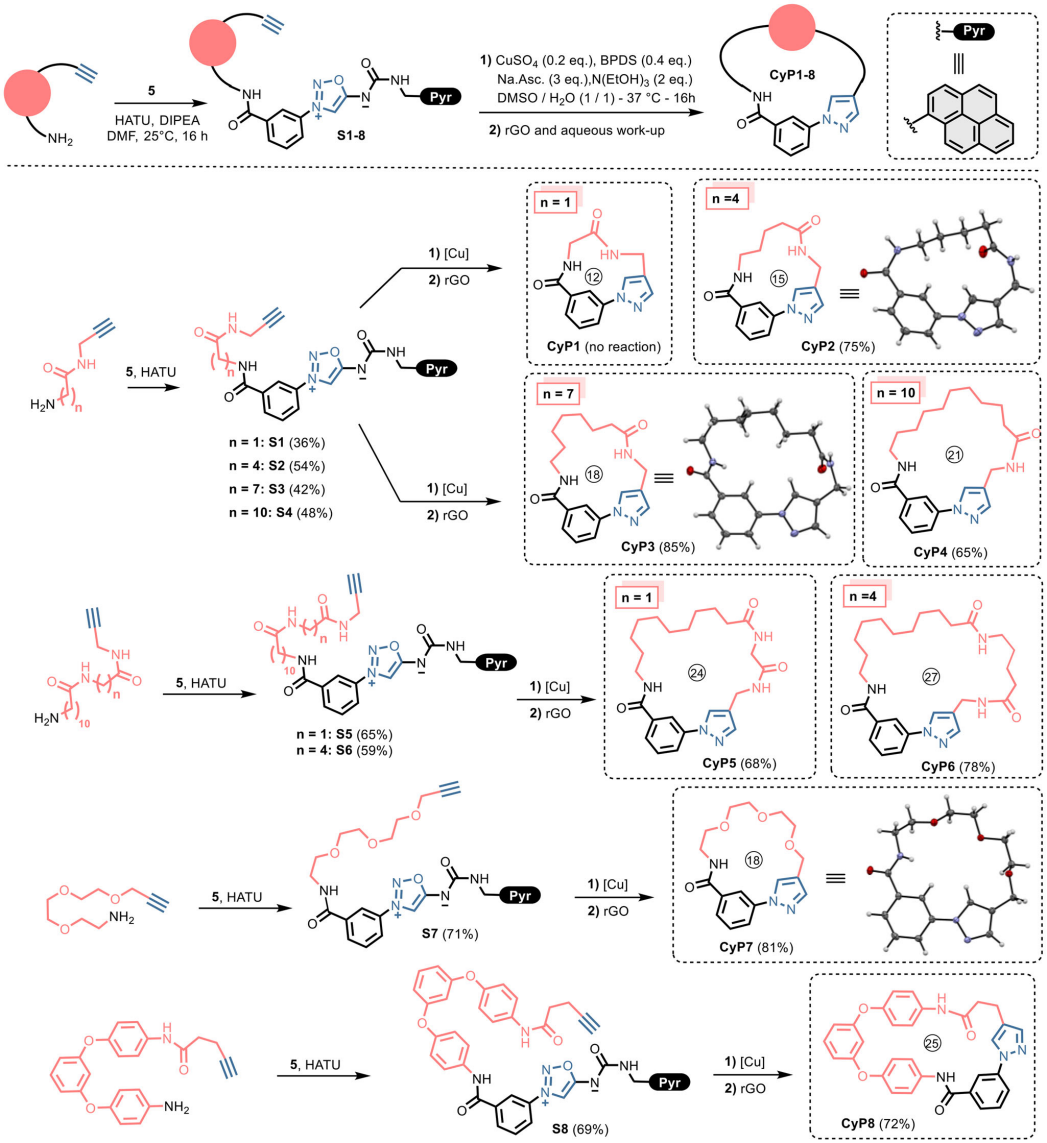
Scope of the reaction. Influence of the size. Reactions were conducted at 35 mM of substrates **S1–8** in DMSO/H_2_O (1:1). Cyclization's were conducted at 37 °C except for **CyP7** which was conducted at 50 °C.

Based on this validation study, we explored the scope of the Cu‐catalyzed cyclization/rGO treatment procedure. To this end, a series of pyrene‐tagged sydnonimine‐alkyne linear substrates was prepared by standard peptide coupling and subjected to the cyclization protocol (Figure 3). We first investigated the influence of ring size on the efficiency of the process.

Alkynylated amines were obtained by standard peptide coupling (see Schemes S2–6 in the supporting information section). As expected, 12‐membered rings could not be obtained due to the strain imposed by the presence of the *N*‐arylpyrazole moiety. Only little degradation of **S1** was observed with no traces of **CyP1** or byproducts due to intermolecular click‐and‐release reaction. However, apart from this anticipated limitation, larger macrocycles **Cy2‐8** were readily synthesized in good yields with no apparent influence of the size of the cycle on the process efficiency. Notably, the crown ether and polyaromatic macrocycles **CyP7** and **CyP8** were obtained in 81% and 72%, respectively. The structures of products **CyP2**, **CyP3,** and **CyP7** were confirmed by X‐rays analysis.

**Figure 4:**
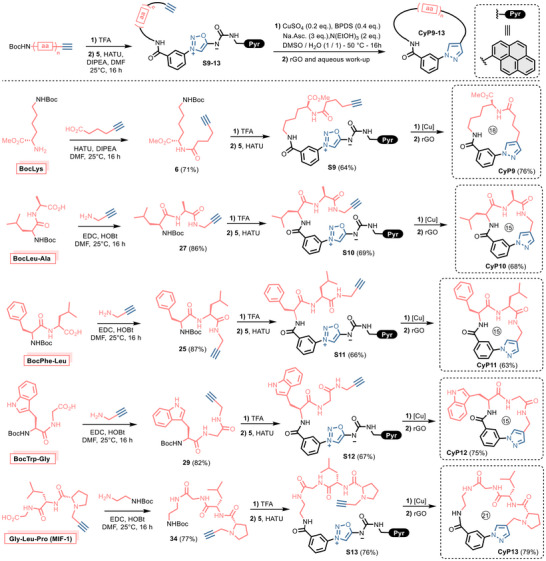
Scope of the reaction. Preparation of cyclic peptides. Reactions were conducted at 35 mM of substrates **S9–13** in DMSO/H_2_O (1:1). Cyclization's were conducted at 50 °C except for **CyP9** which was conducted at 37 °C.

We then successfully applied the procedure to the synthesis of four stapled cyclopeptides (Figure 4). Alkynylated dipeptides **25**, **27,** and **29**, easily obtained using standard peptide synthesis, were successfully coupled to sydnonimine **5** and engaged in the click‐and‐release cyclization step. Cyclic dipeptides **CyP9**‐**12** were obtained in good yields with no apparent influence of the peptide sequence.

The procedure was then applied to the melanocyte‐inhibiting factor (MIF‐1)^[^ ^20^ ^]^ which was successfully converted to the cyclic form **CyP13** in 79% isolated yield. In all cases, the macrocycles were obtained in pure form without the need of additional purification. NMR analysis of **CyP10–13** showed no evidence of diastereoisomer formation, suggesting that no epimerization occurred during the cyclization process. All macrocyclization reactions were run at a substrate concentration of 35 mM. No byproduct resulting from intermolecular reaction was observed in any case. This result can be attributed to the significant difference in reaction kinetics between the inter‐ and intramolecular Cu‐catalyzed click‐and‐release reactions. While the studied scope demonstrated compatibility with ether, indole, ester, and amide functionalities, additional examples are needed to fully assess the chemical function tolerance of the reaction. This will be explored in future work.

## 3 Conclusion

In conclusion, we have developed a technique that combines the use of a Cu‐catalyzed click‐and‐release reaction with pyrene tagging, enabling the straightforward synthesis of macrocycles without the need for extensive purification. Macrocycles can be obtained in good yields in just two steps from alkynylated amine precursors and isolated in pure form using simple filtration and extraction protocols. While this study primarily serves as a proof of concept demonstrating the efficiency of the method, we anticipate that it will be a valuable tool for generating macrocycles libraries.

## 4 Associated Content

### 4.1 Accession Code

CCDC 2424614–2424616 contains the supplementary crystallographic data for this paper. These data can be obtained free of charge via www.ccdc.cam.ac.uk/data_request/cif, or by emailing data_request@ccdc.cam.ac.uk, or by contacting The Cambridge Crystallographic Data Centre, 12 Union Road, Cambridge CB2 1EZ, UK; fax: +44 1223 336033.

## 5 Experimental Section

For details of synthetic procedures and chemical characterizations, see the Supporting Information.

**General procedure** for macrocyclization using intramolecular click‐and‐release:

To a solution of sydnonimine derivative (1 equiv.) and triethanolamine (2 equiv.) in DMSO was added a solution of bathophenanthroline‐disulfonic acid disodium salt hydrate (0.4 equiv.) and CuSO_4_·5H_2_O (0.2 equiv.) in H_2_O. Then a solution of sodium ascorbate (3 equiv.) in H_2_O was added. The solution was stirred at 50 °C for 16 hours and then the solvent was removed. To this mixture was added a solution of rGO previously sonicated in dichloromethane (DCM) (250 mg of rGO for 0.1 mmol of substrate). The dark solution was sonicated for 5 minutes and stirred overnight at room temperature. The solution was then filtrated, the filtrate was washed with water (5 times) and dried over MgSO_4_. The solvent was removed under reduced pressure to afford the pure product without further purification.

## Author Contributions

G. F. optimized the procedure, performed the synthesis of tagged precursors and macrocycles. M. F. developed the synthesis of compound **5** and demonstrated the feasibility of the intramolecular click‐and‐release reaction. D. A. supervised the synthesis of tagged compounds and macrocycles. E. S. and F. T. conceived the idea, acquired funding, guided the experimental work and prepared the manuscript. The manuscript was written through contributions of all authors. All authors have given approval to the final version of the manuscript.

## Conflict of Interests

The authors declare no conflict of interest.

## Supporting information



Supporting Information

## Data Availability

The data that support the findings of this study are available in the supplementary material of this article.
