# Supplementary material for: A Sydnonimine‐based Click‐and‐Release Approach to Cyclic Products
Source: Chemistry. 2025 Apr 21;31(30):e202500860. doi: 10.1002/chem.202500860 (PMC12117170; doi:10.1002/chem.202500860)
Supplement: Supplementary file 1 — Supporting Information [file CHEM-31-e202500860-s001.docx]

Supporting information

A Sydnonimine-based Click-and-Release Approach to Cyclic Products

Guillaume Force,^a^ Minghao Feng,^a^ Davide Audisio,^a^ Pierre Thuery,^b^ Emmanuelle Schulz^c^* and Frédéric Taran^a^*

a. Université Paris Saclay, CEA, INRAE, Département Médicaments et Technologies pour la Santé (DMTS), 91191 Gif-sur-Yvette, France.

b. Université Paris-Saclay, CEA, CNRS, NIMBE, 91191, Gif-sur-Yvette, France.

c. Institut de Chimie Moléculaire et des Matériaux d’Orsay, Université Paris-Saclay, CNRS, 91400 Orsay, France.

E-mail: frederic.taran@cea.fr

E-mail: emmanuelle.schulz@universite-paris-saclay.fr

**Table of contents**

[I. Material and equipment - 3 -](#_Toc135390667)

[II. Synthetic Procedure and analytical Data for compound 5 - 4 -](#_Toc135390668)

[III. Synthetic Procedure and analytical Data for sydnonimines S1-13 -7 -](#_Toc135390669)

[IV. Synthetic Procedure and analytical Data for cyclic compounds CyP2-13 -36 -](#_Toc135390669)

V. NMR Spectra -46

[VI. Crystal structures of compounds CyP2-3 -102 -](#_Toc135390670)

**I. Material and equipment**

All chemical products commercially available were purchased from Sigma-Aldrich, Acros and Fluka and used without further purification. Anhydrous solvents: 1,2-dichloroethane, acetonitrile, DMF, DMSO were purchased in anhydrous form and used without further purification. THF was dried from sodium/benzophenone under nitrogen. Dichloromethane was distilled form calcium hydride under nitrogen.

Reactions were monitored by TLC carried out on silica 0,25 mm (60 F254, Merck) using UV light as visualizing agent and basic aqueous permanganate as developing agent.

^1^H NMR (400 MHz), ^13^C NMR (100 MHz) were measured on a Brucker Avance 400 MHz spectrometer. Chemical shifts are reported in parts per million (ppm) downfield from residual solvents peaks and coupling constants are reported as Hertz (Hz). Splitting patterns are designated as singlet (s), broad singlet (br. s), doublet (d), triplet (t), quartet (q), quintet (quint), heptuplet (hept), multiplet (m). Splitting patterns that could not be interpreted or easily visualized are designated as multiplet (m).

LC-MS mass spectra were recorded using a single quadrupole mass spectrometer (SQD 2, Waters) with electrospray source coupled to Ultra-High Performance Liquid Chromatography (Acquity UPLC H-Class, Waters).

Accurate mass measurements of final compounds were achieved using a high resolution mass spectrometer (Xevo® G2-XS Q-Tof, Waters).

Infrared spectra (IR) were obtained on a Perkin Elmer system 2000 FT-IR spectrophotometer or a Perkin Elmer UATR TWO FTIR spectrophotometer and are reported as wavelength numbers (cm^-1^).

Melting points (Mp) were obtained on a BÜCHI Melting Point B-545 and are reported in °C.

Purification on reversed phase chromatography were done with a Puriflash® XS520Plus (Columns: BIOTAGE® SNAP Cartridge KP-C18-SH-120g or KP-C18-SH-60g).

**II. Synthetic procedure and analytical data for compound 5**

**Scheme S1.** Synthetic route to compound **5**

Methyl 3-((cyanomethyl)amino)benzoate (2)

C_10_H_10_N_2_O_2_

**MW**: 190.20 g.mol^-1^

**Yield**: 75%

Beige solid

To a solution of methyl 3-aminobenzoate **1** (906 mg, 6 mmol, 1 equiv.) in MeCN (8 mL) were added NaI (900 mg, 6 mmol, 1 equiv.), K_2_CO_3_ (829 mg, 6 mmol, 1 equiv.) and chloroacetonitrile (0.4 mL, 6.36 mmol, 1.06 equiv.) The solution was refluxed for 3 days before being filtrated. The filtrate was diluted with AcOEt (5 mL) and H_2_O (5 mL). Then the organic layer was extracted with AcOEt, dried over MgSO_4_ and the solvent was removed under reduced pressure. The crude product was purified by column chromatography (SiO_2_, Hept/AcOEt, 7/3) to afford the desired product as a beige solid (855 mg, 75%).

**^1^H NMR (400 MHz, CDCl_3_)** *δ* 7.53 (d, *J* = 7.6 Hz, 1H), 7.37 (s, 1H), 7.31 (t, *J* = 7.9 Hz, 1H), 6.88 (dd, *J* = 7.9, 1.9 Hz, 1H), 4.35 (t, *J* = 6.3 Hz, 1H), 4.13 (d, *J* = 7.0 Hz, 2H), 3.90 (s, 3H).

**^13^C NMR (101 MHz, CDCl_3_)** *δ* 167.1, 145.3, 131.4, 129.7, 121.1, 118.1, 116.9, 114.2, 52.3, 32.6.

**IR (cm^-1^)** 3366, 2960, 2834, 1702, 1608, 1592, 1524, 1482, 1434, 1280, 1110, 974.

**HRMS** **(ESI-TOF) *m/z*** [M+H]^+^ Calcd for C_10_H_11_N_2_O_2_ 191.0820; Found 191.0819.

**Mp**. 97–99 °C.

3-(3-acetoxyphenyl)-5-amino-1,2,3-oxadiazol-3-ium chloride (3)

C_10_H_10_ClN_3_O_3_

**MW**: 255.66 g.mol^-1^

**Yield**: 87%

Beige solid

To a solution of methyl 3-((cyanomethyl)amino)benzoate **2** (582 mg, 3.06 mmol, 1 equiv.) in dry THF (5 mL) were added *^t^*BuONO (1.090 mL, 9.17 mmol, 3 equiv.) and the mixture was stirred for 3 hours (until TLC showed the fully consumption of starting material) at room temperature. Then the solvent was removed under reduced pressure and the 1.4-dioxane (2 mL) was added following by HCl (4M in 1.4-dioxane) (7.6 mL, 30.6 mmol, 10 equiv.) and the mixture was stirred overnight at room temperature. The solid was filtrated and washed with Et_2_O. The product was dried under reduced pressure to afford the desired product as a pink solid (678 mg, 87%).

**^1^H NMR (400 MHz, DMSO-*d*6)** *δ* 10.13 (s, 2H), 8.80 (s, 1H), 8.54 – 8.47 (m, 1H), 8.38 – 8.30 (m, 2H), 7.98-7.88 (m, 1H), 3.94 (s, 3H).

**^13^C NMR (101 MHz, DMSO-*d*6)** *δ* 169.5, 164.6, 133.7, 133.2, 131.5, 131.1, 127.6, 123.6, 103.1, 52.9.

**IR (cm^-1^)** 3162, 2954, 2873, 1730, 1690, 1585, 1437, 1295, 1266, 1211, 1083, 990.

**HRMS** **(ESI-TOF) *m/z*** [M]^+^ Calcd for C_10_H_10_N_3_O_3_^+^ 220.0722; Found 220.0720.

**Mp**. 195–196 °C.

(3-(3-(methoxycarbonyl)phenyl)-1,2,3-oxadiazol-3-ium-5-yl)((pyren-1-ylmethyl)carbamoyl)amide (4)

C_28_H_20_N_4_O_4_

**MW**: 476.49 g.mol^-1^

**Yield**: 59%

Orange solid

To a solution of triphosgene (51 mg, 173 mmol, 0.33 equiv.), 1-pyrenemethylamine hydrochloride (140 mg, 0.523 mmol, 1 equiv.) in DCM (10 mL) was slowly added a solution of NaHCO_3_ (175 mg, 2.092 mmol, 4 equiv.) in H_2_O (4 mL). The mixture was stirred at 0 °C for 30 minutes. Then 3-(3-acetoxyphenyl)-5-amino-1,2,3-oxadiazol-3-ium chloride **3** ( 133 mg, 0.523 mmol, 1 equiv.) was added and the solution was stirred at room temperature for 5 hours. The reaction was quenched with brine (5 mL), and the DCM was evacued under reduced pressure. The crude was diluted with DCM and the organic layer was extracted with DCM, dried over MgSO_4_ and the solvent was removed under reduced pressure. The crude product was purified by column chromatography (SiO_2_, DCM/MeOH, 100% to 95/05) to afford the desired product as an orange solid (148 mg, 59%).

**^1^H NMR (400 MHz, CDCl_3_)** *δ* 8.26 (d, *J* = 9.2 Hz, 1H), 8.10 – 7.97 (m, 8H), 7.91 – 7.83 (m, 3H), 7.58 (d, *J* = 7.2 Hz, 1H), 7.40 (t, *J* = 7.9 Hz, 1H), 6.89 (t, *J* = 5.5 Hz, 1H), 5.14 (d, *J* = 5.5 Hz, 2H), 3.92 (s, 3H).

**^13^C NMR (101 MHz, CDCl_3_)** *δ* 172.7, 164.6, 161.5, 133.7, 133.1, 132.5, 132.1, 131.1, 130.7, 130.6, 130.2, 128.67, 127.6, 127.4, 127.0, 126.4, 125.8, 125.0, 125.0, 124.7, 124.7, 124.7, 124.6, 123.0, 121.8, 101.6, 52.8, 42.8.

**IR (cm^-1^)** 3266, 2973, 1720, 1632, 1586, 1524, 1487, 1434, 1267, 1183, 1045, 956.

**HRMS** **(ESI-TOF) *m/z*** [M+H]^+^ Calcd for C_28_H_21_N_4_O_4_ 477.1563; Found 477.1569.

**Mp**. 167–169 °C.

(3-(3-carboxyphenyl)-1,2,3-oxadiazol-3-ium-5-yl)((pyren-1-ylmethyl)carbamoyl)amide (5)

C_27_H_18_N_4_O_4_

**MW**: 462.47 g.mol^-1^

**Yield**: 89%

Yellow solid

To a solution of (3-(3-(methoxycarbonyl)phenyl)-1,2,3-oxadiazol-3-ium-5-yl)((pyren-1-ylmethyl)carbamoyl)amide **4** (30 mg, 0.063 mmol, 1 equiv.) in a mixture THF/MeOH (1 mL : 1 mL) was added a solution of NaOH (12.5 mg, 0.315 mmol, 5 equiv.) in H_2_O (1 mL). The mixture was stirred 1 hour at room temperature. The mixture was diluted with H_2_O and acidified with HCl (1M) until pH = 2,. Then AcOEt was added and the organic layer was extracted, and the solvent was removed under reduced pressure to afford the product as a yellow solid without further purification (27 mg, 89%).

**^1^H NMR (400 MHz, DMSO-*d*6)** *δ* 13.38 (brs, 1H), 8.56-8.45 (m, 2H), 8.45-8.39 (m, 1H), 8.34-8.21 (m, 6H), 8.15 (s, 2H), 8.10-8.05 (m, 2H), 7.87-7.77 (m, 2H), 5.02 (d, *J* = 6.0 Hz, 2H).

**^13^C NMR**, the solubility of the product is very low, ^13^C NMR analysis could not be perform.

**IR (cm^-1^)** 3162, 2953, 2874, 1730, 1691, 1585, 1437, 1295, 1211, 1104, 1083, 990.

**HRMS** **(ESI-TOF) *m/z*** [M+H]^+^ Calcd for C_27_H_19_N_4_O_4_ 463.1406; Found 463.1406.

**Mp**. 238–240 °C.

## III. Synthetic procedure and analytical data for sydnonimines S1-13

**General procedures:**

**General procedure A for *N*-protecting step of amino acid.**

To a solution of amino acid (1 equiv.) in MeOH, was added Boc_2_O (1 equiv.) and triethylamine (1.2 equiv.) and the solution was stirred overnight at 60 °C. Then the solvent was removed under reduced pressure, and the mixture was redilluted with AcOEt, this organic layer was washed with HCl (0.25 M, 3x5 mL), and dried with brine, over MgSO_4_ and the solvent was removed under reduced pressure, to afford the desired product without further purification.

**General procedure B for peptide bond coupling**

To a solution of acid (1 equiv.) in DMF, were added HATU (1.5 equiv.) and DIPEA (3 equiv.). The reaction was stirred at room temperature for 20 minutes and amine or amine hydrochloride (1.5 equiv.) was added. After 4 hours the reaction was quenched by NH_4_Cl_sat_. The organic layer was extracted with AcOEt, dried over MgSO_4_ and the solvent was removed under reduced pressure. The crude product was purified by column chromatography with the indicated eluent to afford the desired product.

**General procedure C for peptide bond coupling**

To a solution of acid (1 equiv.), amine (1 or 1.2 equiv.) and HOBt (1.2 equiv.) in DMF, were added EDC hydrochloride (1.2 equiv.) and DIPEA (2.5 equiv.). The reaction was stirred at room temperature overnight. Then the mixture was concentrated and the crude product was purified by column chromatography with the indicated eluent to afford the desired product.

**Synthesis of precursors 6-16**

**Scheme S2.** Synthesis of precursors **6-16**

Methyl N^6^-(tert-butoxycarbonyl)-N^2^-(hex-5-ynoyl)-L-lysinate (6)

C_18_H_30_N_2_O_5_

**MW**: 354.45 g.mol^-1^

**Yield**: 71%

Colorless oil

Following Generale Procedure **B**, using hex-5-ynoic acid (190 µL, 1.73 mmol, 1 equiv.), DMF (5 mL), *H*-Lys(Boc)-OMe hydrochloride (769 mg, 2.59 mmol, 1.5 equiv.), HATU (985 mg, 2.59 mmol, 1.5 equiv.) and DIPEA (879 µL, 5.18 mmol, 3 equiv.) The crude product was purified by column chromatography (SiO_2_, Hept/AcOEt, 70/30) to afford the desired product as a colorless oil (435 mg, 71%).

**^1^H NMR (400 MHz, MeOD)** *δ* 4.46 – 4.35 (m, 1H), 3.70 (s, 3H), 3.03 (t, *J* = 6.7 Hz, 2H), 2.38 (t, *J* = 7.5 Hz, 2H), 2.24 – 2.17 (m, 3H), 1.88 – 1.74 (m, 3H), 1.73 – 1.65 (m, 1H), 1.41 (s, 9H), 1.51 – 1.32 (m, 4H), NH unobserved.

**^13^C NMR (101 MHz, MeOD)** *δ* 175.8, 174.1, 158.4, 84.0, 79.9, 70.2, 53.6, 52.7, 40.8, 35.4, 31.8, 30.2, 28.7, 25.7, 23.9, 18.4.

**IR (cm^-1^)** 3310, 2958, 2938, 2851, 1733, 1670, 1638, 1540, 1452, 1369, 1186, 1134, 836.

**HRMS** **(ESI-TOF) *m/z*** [M+Na]^+^ Calcd for C_18_H_30_N_2_O_5_Na 377.2052; Found 377.2050.

(tert-butoxycarbonyl)glycine (9)

C_7_H_13_NO_4_

**MW**: 175.18 g.mol^-1^

Yield: 81%

White solid

Following Procedure **A**, using glycine (400 mg, 5.33 mmol, 1 equiv.), Boc_2_O (1.16 g, 5.33 mmol, 1 equiv.), Et_3_N (891 µL, 6.39 mmol, 1.2 equiv.) and MeOH (15 mL). The product was obtained as a white solid (755 mg, 81%).

**^1^H NMR (400 MHz, CDCl_3_)** *δ* 10.80 (s, 1H), 5.42 (t, *J* = 5.1 Hz, 1H), 3.85 (d, *J* = 5.5 Hz, 2H), 1.35 (s, 9H).

**^13^C NMR (101 MHz, CDCl_3_)** *δ* 171.7, 156.2, 80.3, 42.2, 28.3.

5-((tert-butoxycarbonyl)amino)pentanoic acid (10)

C_10_H_19_NO_4_

**MW**: 217.26 g.mol^-1^

**Yield**: 89%

White solid

Following Procedure **A**, using 5-aminopentanoic acid (400 mg, 3.41 mmol, 1 equiv.), Boc_2_O (745 mg, 3.41 mmol, 1 equiv.), Et_3_N (570 µL, 4.09 mmol, 1.2 equiv.) and MeOH (15 mL). The product was obtained as a white solid (659 mg, 89%).

**^1^H NMR (400 MHz, MeOD)** *δ* 3.05 (t, *J* = 6.8 Hz, 2H), 2.31 (t, *J* = 7.3 Hz, 2H), 1.66 – 1.56 (m, 2H), 1.54 – 1.46 (m, 2H), 1.43 (s, 9H), COOH and NH unobserved.

**^13^C NMR (101 MHz, MeOD)** *δ* 177.4, 158.5, 79.8, 40.9, 34.5, 30.4, 28.8, 23.3.

**IR (cm^-1^)** 3259, 2950, 2865, 1679, 1658, 1416, 1160, 1022..

**HRMS** **(ESI-TOF) *m/z*** [M-H]^-^ Calcd for C_10_H_18_NO_4_ 216.1236; Found 216.1239.

**Mp**. 105–107 °C.

8-((tert-butoxycarbonyl)amino)octanoic acid (11)

C_13_H_25_NO_4_

**MW**: 259.35 g.mol^-1^

**Yield**: 92%

White solid

Following Procedure **A**, using 8-aminooctanoic acid (400 mg, 2.51 mmol, 1 equiv.), Boc_2_O (547 mg, 2.51 mmol, 1 equiv.), Et_3_N (419 µL, 3.01 mmol, 1.2 equiv.) and MeOH (15 mL). The product was obtained as a white solid (597 mg, 92%).

**^1^H NMR (400 MHz, MeOD)** *δ* 3.02 (t, *J* = 7.0 Hz, 2H), 2.28 (t, *J* = 7.4 Hz, 2H), 1.60 (p, *J* = 7.3 Hz, 2H), 1.52 – 1.40 (m, 2H), 1.43 (s, 9H), 1.39 – 1.27 (m, 6H), COOH and NH unobserved.

**^13^C NMR (101 MHz, MeOD)** *δ* 177.6, 158.5, 79.8, 41.3, 34.9, 30.9, 30.1, 30.1, 28.8, 27.7, 26.0.

**IR (cm^-1^)** 3260, 2949, 2862, 1681, 1655, 1426, 1359, 1256, 1026.

**HRMS** **(ESI-TOF) *m/z*** [M-H]^-^ Calcd for C_13_H_24_NO_4_ 258.1705; Found 258.1707.

**Mp**. 106–108 °C.

11-((tert-butoxycarbonyl)amino)undecanoic acid (12)

C_16_H_31_NO_4_

**MW**: 301.43 g.mol^-1^

**Yield**: 86%

White solid

Following Procedure **A**, using 11-aminoundecanoic acid (500 mg, 2.48 mmol, 1 equiv.), Boc_2_O (541 mg, 2.48 mmol, 1 equiv.), Et_3_N (415 µL, 2.98 mmol, 1.2 equiv.) and MeOH (18 mL). The product was obtained as a white solid (645 mg, 86%).

**^1^H NMR (400 MHz, MeOD)** *δ* 3.01 (t, *J* = 7.0 Hz, 2H), 2.28 (t, *J* = 7.4 Hz, 2H), 1.66 – 1.54 (m, 2H), 1.51 – 1.38 (m, 2H), 1.43 (s, 9H), 1.37 – 1.26 (m, 12H)n NH and COOH unobserved.

**^13^C NMR (101 MHz, MeOD)** *δ* 177.7, 158.6, 79.8, 41.4, 34.9, 31.0, 30.6, 30.5, 30.4 (2C), 30.2, 28.8, 27.8, 26.1.

**IR (cm^-1^)** 3269, 2950, 2872, 1687, 1651, 1530, 1431, 1259, 1149.

**HRMS** **(ESI-TOF) *m/z*** [M-H]^-^ Calcd for C_16_H_30_NO_4_ 300.2175; Found 300.2176.

**Mp**. 110–112 °C.

Tert-butyl (2-oxo-2-(prop-2-yn-1-ylamino)ethyl)carbamate (13)

C_10_H_16_N_2_O_3_

**MW**: 212.25 g.mol^-1^

**Yield**: 52%

White solid

Following Procedure **B,** using **9** (275 mg, 1.57 mmol, 1 equiv.), propargylamine (150 µL, 2.35 mmol, 1.5 equiv.), HATU (893 mg, 2.35 mmol, 1.5 equiv.), DIPEA (656 µL, 4.71 mmol, 3 equiv.) and DMF (8 mL). The crude product was purified by column chromatography (SiO_2_, Hept/AcOEt, 70/30) to afford the desired product as a yellow solid (173 mg, 52%).

**^1^H NMR (400 MHz, MeOD)** *δ* 3.98 (d, *J* = 2.4 Hz, 2H), 3.70 (s, 2H), 2.57 (t, *J* = 2.5 Hz, 1H), 1.45 (s, 9H), NH unobserved.

**^13^C NMR (101 MHz, MeOD)** *δ* 172.1, 158.3, 80.7, 80.5, 72.1, 44.5, 29.4, 28.7.

**IR (cm^-1^)** 3085, 2948, 2868, 1682, 1658, 1411, 1250, 1158.

**HRMS** **(ESI-TOF) *m/z*** [M+Na]^+^ Calcd for C_10_H_16_N_2_O_3_Na 235.1059; Found 235.1057.

**Mp**. 102–104 °C.

Tert-butyl (5-oxo-5-(prop-2-yn-1-ylamino)pentyl)carbamate (14)

C_13_H_22_N_2_O_3_

**MW**: 254.33 g.mol^-1^

**Yield**: 78%

Yellow solid

Following Procedure **B**, using **10** (317 mg, 1.46 mmol, 1 equiv.), propargylamine (140 µL, 2.19 mmol, 1.5 equiv.), HATU (832 mg, 2.19 mmol, 1.5 equiv.), DIPEA (697 µL, 4.38 mmol, 3 equiv.) and DMF (8 mL). The crude product was purified by column chromatography (SiO_2_, Hept/AcOEt, 70/30) to afford the desired product as a yellow solid (289 mg, 78%).

**^1^H NMR (400 MHz, CDCl_3_)** *δ* 6.71 (s, 1H), 4.88 (s, 1H), 3.93 (dd, *J* = 5.3, 2.6 Hz, 2H), 3.03 (dd, *J* = 12.7, 6.3 Hz, 2H), 2.21 – 2.13 (m, 3H), 1.64 – 1.54 (m, 2H), 1.43 (dt, *J* = 14.0, 7.0 Hz, 2H), 1.35 (s, 9H).

**^13^C NMR (101 MHz, CDCl_3_)** *δ* 172.9, 156.2, 79.9, 79.0, 71.2, 39.8, 35.5, 29.4, 28.9, 28.4, 22.6.

**IR (cm^-1^)** 3313, 2932, 2873, 1683, 1653, 1525, 1364, 1278, 1151.

**HRMS** **(ESI-TOF) *m/z*** [M+Na]^+^ Calcd for C_13_H_22_N_2_O_3_Na 277.1528; Found 277.1526.

**Mp**. 95–97 °C.

Tert-butyl (8-oxo-8-(prop-2-yn-1-ylamino)octyl)carbamate (15)

C_16_H_28_N_2_O_3_

**MW**: 296.41 g.mol^-1^

**Yield**: 82%

Yellow solid

Following Procedure **B**, using **11** (348 mg, 1.343 mmol, 1 equiv.), propargylamine (129 µL, 2.014 mmol, 1.5 equiv.), HATU (765 mg, 2.014 mmol, 1.5 equiv.), DIPEA (641 µL, 4.029 mmol, 3 equiv.) and DMF (8 mL). The crude product was purified by column chromatography (SiO_2_, Hept/AcOEt, 70/30) to afford the desired product as a yellow solid (325 mg, 82%).

**^1^H NMR (400 MHz, MeOD)** *δ* 3.93 (d, *J* = 2.5 Hz, 2H), 3.01 (t, *J* = 7.0 Hz, 2H), 2.55 (t, *J* = 2.5 Hz, 1H), 2.19 (t, *J* = 7.5 Hz, 2H), 1.67 – 1.56 (m, 2H), 1.50 – 1.39 (m, 2H), 1.43 (s, 9H), 1.36 – 1.27 (m, 6H), NH unobserved.

**^13^C NMR (101 MHz, MeOD)** *δ* 175.9, 158.6, 80.6, 79.8, 72.0, 41.3, 36.7, 30.8, 30.05, 29.98, 29.29, 28.7, 27.6, 26.7.

**IR (cm^-1^)** 3329, 2931, 2865, 1682, 1650, 1530, 1250, 1158.

**HRMS** **(ESI-TOF) *m/z*** [M+Na]^+^ Calcd for C_16_H_28_N_2_O_3_Na 319.1998; Found 319.1997.

**Mp**. 96–98 °C.

Tert-butyl (11-oxo-11-(prop-2-yn-1-ylamino)undecyl)carbamate (16)

C_19_H_34_N_2_O_3_

**MW**: 338.49 g.mol^-1^

**Yield**: 97%

Yellow solid

Following Procedure **B**, using **12** (300 mg, 0.996 mmol, 1 equiv.), propargylamine (95 µL, 1.49 mmol, 1.5 equiv.), HATU (566 mg, 1.49 mmol, 1.5 equiv.), DIPEA (505 µL, 2.98 mmol, 3 equiv.) and DMF (8 mL). The crude product was purified by column chromatography (SiO_2_, Hept/AcOEt, 70/30) to afford the desired product as a yellow solid (327 mg, 97%).

**^1^H NMR (400 MHz, CDCl_3_)** *δ* 5.68 (s, 1H), 4.49 (s, 1H), 4.05 (dd, *J* = 5.0, 2.5 Hz, 2H), 3.14–3.04 (m, 2H), 2.22 (t, *J* = 2.5 Hz, 1H), 2.18 (t, *J* = 7.8 Hz, 2H), 1.67–1.58 (m, 2H), 1.51–1.37 (m, 2H), 1.44 (s, 9H), 1.27–1.22 (m, 12H).

**^13^C NMR (101 MHz, CDCl_3_)** *δ* 172.9, 156.2, 79.8, 77.4, 71.6, 40.8, 36.6, 30.2, 29.5, 29.4, 29.35, 29.32 (2C), 29.28, 28.6, 26.9, 25.6.

**IR (cm^-1^)** 3315, 3298, 2979, 2914, 1848, 2120, 1683, 1654, 1524, 1455, 1365, 1281, 1240, 1168, 939, 884, 789.

**HRMS** **(ESI-TOF) *m/z*** [M+Na]^+^ Calcd for C_19_H_34_N_2_O_3_Na 361.2467; Found 361.2470.

**Mp**. 98–100 °C.

**Synthesis of precursors 17-23**

**Scheme S3.** Synthesis of precursors **17-23**

Methyl 5-aminopentanoate (17)

C_6_H_13_NO_2_

**MW**: 131.17 g.mol^-1^

**Yield**: 98%

White solid

To a solution of 5-aminopentanoic acid (1 g, 8.54 mmol, 1 equiv.) in MeOH (10 mL) was added at 0 °C thionyl chloride (933 µL, 12.8 mmol, 1.5 equiv.) and the solution was stirred for 12 hours at room temperature. Then the solvent was removed under reduced pressure to aford the product without further purification (1.09 g, 98%).

**^1^H NMR (400 MHz, MeOD)** *δ* 3.67 (s, 3H), 3.03–2.85 (m, 2H), 2.47–2.32 (m, 2H), 1.79–1.58 (m, 4H), NH_2_ unobserved.

**^13^C NMR (101 MHz, MeOD)** *δ* 175.2, 52.1, 40.4, 33.9, 27.9, 22.7.

The spectral data (^1^H-NMR) and (^13^C-NMR) was consistent with reported one.^[[1]](#footnote-1)^

Methyl (11-((tert-butoxycarbonyl)amino)undecanoyl)glycinate (18)

C_19_H_36_N_2_O_5_

**MW**: 372.51 g.mol^-1^

**Yield**: 64%

White solid

Following Procedure **B**, using *N*-Boc-undecanoic acid (300 mg, 0.996 mmol, 1 equiv.), glycine methyl ester hydrochloride (187 mg, 1.49 mmol, 1.5 equiv.), HATU (566 mg, 1.49 mmol, 1.5 equiv.), DIPEA (505 µL, 2.98 mmol, 3 equiv.) and DMF (8 mL). The crude product was purified by column chromatography (SiO_2_, Hept/AcOEt, 70/30) to afford the desired product as a yellow solid (238 mg, 64%).

**^1^H NMR (400 MHz, MeOD)** *δ* 3.92 (s, 2H), 3.71 (s, 3H), 3.02 (t, *J* = 7.0 Hz, 2H), 2.25 (t, *J* = 7.5 Hz, 2H), 1.71–1.57 (m, 2H), 1.43 (s, 9H), 1.50–1.42 (m, 2H), 1.36–1.28 (m, 12H), NH unobserved.

**^13^C NMR (101 MHz, MeOD)** *δ* 176.6, 171.8, 158.4, 79.6, 52.5, 41.8, 41.3, 36.7, 30.9, 30.6, 30.5, 30.4 (2C), 30.1, 28.8, 27.8, 26.8.

**IR (cm^-1^)** 3351, 3298, 2941, 2920, 2847, 1758, 1673, 1541, 1518, 1459, 1412, 1368, 1252, 1202, 1158, 1053, 985, 852.

**HRMS** **(ESI-TOF) *m/z*** [M+Na]^+^ Calcd for C_19_H_36_N_2_O_5_Na 395.2522; Found 395.2520.

**Mp**. 61–63 °C.

Methyl 5-(11-((tert-butoxycarbonyl)amino)undecanamido)pentanoate (19)

C_22_H_42_N_2_O_5_

**MW**: 414.59 g.mol^-1^

**Yield**: 75%

White solid

Following Procedure **B**, using **17** (300 mg, 0.996 mmol, 1 equiv.), *N*-Boc-undecanoic acid (195 mg, 1.49 mmol, 1.5 equiv.), HATU (566 mg, 1.49 mmol, 1.5 equiv.), DIPEA (505 µL, 2.98 mmol, 3 equiv.) and DMF (8 mL). The crude product was purified by column chromatography (SiO_2_, Hept/AcOEt, 70/30) to afford the desired product as a yellow solid (309 mg, 75%).

**^1^H NMR (400 MHz, MeOD)** *δ* 3.65 (s, 3H), 3.17 (t, *J* = 6.8 Hz, 2H), 3.01 (t, *J* = 7.0 Hz, 2H), 2.35 (t, *J* = 7.3 Hz, 2H), 2.17 (t, *J* = 7.5 Hz, 2H), 1.69–1.57 (m, 4H), 1.56–1.47 (m, 2H), 1.48–1.37 (m, 2H), 1.43 (s, 9H), 1.37–1.26 (s, 12H), NH unobserved.

**^13^C NMR (101 MHz, MeOD)** *δ* 176.1, 175.6, 158.4, 79.7, 52.0, 41.3, 39.7, 37.1, 34.3, 30.9, 30.5, 30.4, 30.3 (2C), 30.2, 29.7, 28.8, 27.8, 27.0, 23.2.

**IR (cm^-1^)** 3355, 3330, 2939, 2918, 2851, 1736, 1679, 1638, 1523, 1283, 1249, 1165, 1008, 837.

**HRMS** **(ESI-TOF) *m/z*** [M+Na]^+^ Calcd for C_22_H_42_N_2_O_5_Na 437.2991; Found 437.2989.

**Mp**. 63–65 °C.

(11-((tert-butoxycarbonyl)amino)undecanoyl)glycine (20)

C_18_H_34_N_2_O_5_

**MW**: 358.48 g.mol^-1^

**Yield**: 92%

White solid

To a solution of **18** (292 mg, 0.782 mmol, 1 equiv.) in a mixture THF/MeOH (4 mL : 4 mL) was added a solution of NaOH (125 mg, 3.13 mmol, 4 equiv.) in H_2_O (4 mL). The mixture was stirred 1 hour at room temperature. The mixture was diluted with H_2_O and AcOEt and the aqueous layer was extracted and acidified with HCl (1M) until pH = 2. Then AcOEt was added and the organic layer was extracted, dried over MgSO_4_ and the solvent was removed under reduced pressure to afford the product as a white solid without further purification (257 mg, 92%).

**^1^H NMR (400 MHz, MeOD)** *δ* 3.88 (s, 2H), 3.01 (t, *J* = 7.0 Hz, 2H), 2.24 (t, *J* = 7.5 Hz, 2H), 1.68–1.56 (m, 2H), 1.50–1.47 (m, 2H), 1.43 (s, 9H), 1.36–1.27 (m, 12H), NH and OH unobserved.

**^13^C NMR (101 MHz, MeOD)** *δ* 176.5, 173.0, 158.4, 79.6, 41.7, 41.3, 36.7, 30.9, 30.5, 30.4, 30.3 (2C), 30.1, 28.8, 27.8, 26.7.

**IR (cm^-1^)** 3315, 2918, 2849, 1680, 1635, 1522, 1465, 1414, 1365, 1281, 1162, 1114, 855.

**HRMS** **(ESI-TOF) *m/z*** [M+Na]^+^ Calcd for C_18_H_34_N_2_O_5_Na 381.2365; Found 381.2367.

**Mp**. 105–107 °C.

5-(11-((tert-butoxycarbonyl)amino)undecanamido)pentanoic acid (21)

C_21_H_40_N_2_O_5_

**MW**: 400.56 g.mol^-1^

**Yield**: 94%

White solid

To a solution of **19** (315 mg, 0.760 mmol, 1 equiv.) in a mixture THF/MeOH (4 mL : 4 mL) was added a solution of NaOH (121 mg, 3.04 mmol, 4 equiv.) in H_2_O (4 mL). The mixture was stirred 1 hour at room temperature. The mixture was diluted with H_2_O and AcOEt and the aqueous layer was extracted and acidified with HCl (1M) until pH = 2. Then AcOEt was added and the organic layer was extracted, dried over MgSO_4_ and the solvent was removed under reduced pressure to afford the product as a white solid without further purification (285 mg, 94%).

**^1^H NMR (400 MHz, MeOD)** *δ* 3.18 (t, *J* = 6.8 Hz, 2H), 3.01 (t, *J* = 7.0 Hz, 2H), 2.31 (t, *J* = 7.2 Hz, 2H), 2.17 (t, *J* = 7.5 Hz, 2H), 1.67–1.50 (m, 6H), 1.43 (s, 9H), 1.49–1.38 (m, 2H), 1.39–1.25 (m, 12H), NH and OH unobverved.

**^13^C NMR (101 MHz, MeOD)** *δ* 177.2, 176.3, 158.5, 79.8, 41.4, 39.9, 37.1, 34.5, 31.0, 30.6, 30.5, 30.4 (2C), 30.3, 29.9, 28.8, 27.8, 27.1, 23.3.

**IR (cm^-1^)** 3350, 3328, 2936, 2917, 2851, 1698, 1678, 1528, 1479, 1415, 1283, 1252, 1170, 1120, 1008, 924.

**HRMS** **(ESI-TOF) *m/z*** [M+Na]^+^ Calcd for C_21_H_40_N_2_O_5_Na 423.2835; Found 423.2833.

**Mp**. 112–114 °C.

Tert-butyl (11-oxo-11-((2-oxo-2-(prop-2-yn-1-ylamino)ethyl)amino)undecyl)carbamate (22)

C_21_H_37_N_3_O_4_

**MW**: 395.54 g.mol^-1^

**Yield**: 73%

Yellow solid

Following Procedure **B**, using **20** (295 mg, 0.823 mmol, 1 equiv.), propargylamine (78 µL, 1.23 mmol, 1.5 equiv.), HATU (469 mg, 1.23 mmol, 1.5 equiv.), DIPEA (393 µL, 2.47 mmol, 3 equiv.) and DMF (8 mL). The crude product was purified by column chromatography (SiO_2_, DCM/MeOH, 95/05) to afford the desired product as a yellow solid (237 mg, 73%).

**^1^H NMR (400 MHz, MeOD)** *δ* 3.98 (d, *J* = 2.0 Hz, 2H), 3.84 (s, 2H), 3.01 (t, *J* = 7.0 Hz, 2H), 2.57 (t, *J* = 2.5 Hz, 1H), 2.27 (t, *J* = 7.6 Hz, 2H), 1.69 – 1.56 (m, 2H), 1.47 – 1.38 (m, 11H), 1.35 – 1.21 (m, 12H), NH unobserved.

**^13^C NMR (101 MHz, MeOD)** *δ* 177.0, 171.5, 158.6, 80.4, 79.8, 72.3, 43.4, 41.4, 36.9, 31.0, 30.7, 30.5, 30.4, 30.4, 30.3, 29.5, 28.8, 27.9, 26.7.

**IR (cm^-1^)** 3373, 3303, 2921, 2851, 1681, 1655, 1639, 1543, 1519, 1243, 1204, 1171, 1136, 1025, 840.

**HRMS** **(ESI-TOF) *m/z*** [M+Na]^+^ Calcd for C_21_H_37_N_3_O_4_Na 418.2682; Found 418.2680.

**Mp**. 109–111 °C.

Tert-butyl (11-oxo-11-((5-oxo-5-(prop-2-yn-1-ylamino)pentyl)amino)undecyl)carbamate (23)

C_24_H_43_N_3_O_4_

**MW**: 437.62 g.mol^-1^

**Yield**: 74%

Yellow solid

Following Procedure **B**, using **21** (126 mg, 0.315 mmol, 1 equiv.), propargylamine (30 µL, 0.472 mmol, 1.5 equiv.), HATU (179 mg, 0.472 mmol, 1.5 equiv.), DIPEA (164 µL, 0.945 mmol, 3 equiv.) and DMF (8 mL). The crude product was purified by column chromatography (SiO_2_, DCM/MeOH, 95/05) to afford the desired product as a yellow solid (107 mg, 74%).

**^1^H NMR (400 MHz, MeOD)** *δ* 3.95 (d, *J* = 2.5 Hz, 2H), 3.17 (t, *J* = 6.8 Hz, 2H), 3.01 (t, *J* = 7.0 Hz, 2H), 2.56 (t, *J* = 2.5 Hz, 1H), 2.21 (t, *J* = 7.3 Hz, 2H), 2.16 (t, *J* = 7.5 Hz, 2H), 1.69 – 1.55 (m, 4H), 1.55 – 1.40 (m, 4H), 1.43 (s, 9H), 1.36 – 1.22 (m, 12H), NH unobserved.

**^13^C NMR (101 MHz, MeOD)** *δ* 176.3, 175.5, 158.6, 80.7, 79.8, 72.1, 41.4, 39.9, 37.2, 36.2, 31.0, 30.64, 30.55, 30.4 (2C), 30.3, 29.8, 29.4, 28.8, 27.9, 27.1, 24.1.

**IR (cm^-1^)** 3319, 2940, 2918, 2851, 1677, 1631, 1525, 1417, 1281, 1249, 1168.

**HRMS** **(ESI-TOF) *m/z*** [M+H]^+^ Calcd for C_24_H_44_N_3_O_4_ 438.3332; Found 438.3333.

**Mp**. 102–104 °C.

**Synthesis of precursors 24-29**

**Scheme S4.** Synthesis of precursors **24-29**

(tert-butoxycarbonyl)-L-phenylalanyl-L-leucine (24)

C_20_H_30_N_2_O_5_

**MW**: 378.47 g.mol^-1^

**Yield**: 78%

White solid

Following Procedure **A**, using ***L*-Phenylalanyl-*L*-leucine** (500 mg, 1.79 mmol, 1 equiv.), Boc_2_O (391 mg, 1.79 mmol, 1 equiv.), Et_3_N (301 µL, 2.16 mmol, 1.2 equiv.) and MeOH (15 mL). The product was obtained as a white solid (528 mg, 78%).

**^1^H NMR (400 MHz, MeOD)** *δ* 7.33 – 7.22 (m, 4H), 7.21 – 7.15 (m, 1H), 4.48 (t, *J* = 7.3 Hz, 1H), 4.37 (dd, *J* = 9.3, 4.8 Hz, 1H), 3.13 (dd, *J* = 13.9, 4.8 Hz, 1H), 2.81 (dd, *J* = 13.8, 9.6 Hz, 1H), 1.80 – 1.58 (m, 3H), 1.35 (s, *J* = 13.5 Hz, 9H), 0.93 (dd, *J* = 10.5, 6.4 Hz, 6H), COOH and NH unobserved.

**^13^C NMR (101 MHz, MeOD)** *δ* 175.6, 174.3, 157.5, 138.6, 130.4, 129.3, 127.6, 80.5, 57.0, 52.0, 41.8, 39.1, 28.6, 25.8, 23.4, 21.9.

**IR (cm^-1^)** 3330, 3297, 2953, 2929, 2870, 1670, 1636, 1526, 1300, 1249, 1170.

**HRMS** **(ESI-TOF) *m/z*** [M-H]^-^ Calcd for C_20_H_29_N_2_O_5_ 377.2076; Found 377.2077.

**Mp**. 152–154 °C.

Tert-butyl ((S)-1-(((S)-4-methyl-1-oxo-1-(prop-2-yn-1-ylamino)pentan-2-yl)amino)-1-oxo-3-phenylpropan-2-yl)carbamate (25)

C_23_H_33_N_3_O_4_

**MW**: 415.54 g.mol^-1^

**Yield**: 87%

Yellow solid

Following Procedure **C**, using (*tert*-butoxycarbonyl)-*L*-phenylalanyl-*L*-leucine **24** (204 mg, 0.539 mmol, 1 equiv.), propargylamine (41 µL, 0.647 mmol, 1.2 equiv.), HOBt (87 mg, 0.647 mmol, 1.2 equiv.), EDC hydrochloride (124 mg, 0.647 mmol, 1.2 equiv.), DIPEA (220 µL, 1.347 mmol, 2.5 equiv.) and DMF (8 mL). The crude product was purified by column chromatography (SiO_2_, DCM/MeOH, 95/05) to afford the desired product as a yellow solid (195 mg, 87%).

**^1^H NMR (400 MHz, CD_2_Cl_2_)** *δ* 7.34 – 7.29 (m, 2H), 7.28 – 7.24 (m, 1H), 7.23 – 7.18 (m, 2H), 6.80 (s, 1H), 6.51 (d, *J* = 8.1 Hz, 1H), 5.14 (d, *J* = 6.8 Hz, 1H), 4.48 – 4.39 (m, 1H), 4.38 – 4.27 (m, 2H), 3.10 (dd, *J* = 13.9, 6.2 Hz, 1H), 2.99 (dd, *J* = 13.9, 7.5 Hz, 1H), 2.24 (t, *J* = 2.4 Hz, 1H), 1.68 (ddd, *J* = 13.5, 8.5, 5.0 Hz, 1H), 1.58 – 1.44 (m, 2H), 1.40 (s, 9H), 0.89 (dd, *J* = 6.3, 4.4 Hz, 6H), one NH unobserved.

**^13^C NMR (101 MHz, CD_2_Cl_2_)** *δ* 171.9, 171.7, 156.3, 136.9, 129.7, 129.1, 127.4, 80.9, 80.1, 71.3, 56.6, 52.0, 41.0, 38.1, 29.3, 28.4, 25.0, 23.2, 21.8.

**IR (cm^-1^)** 3324, 3297, 2953, 2929, 2870, 1686, 1637, 1526, 1447, 1291, 1249, 1170.

**HRMS** **(ESI-TOF) *m/z*** [M+Na]^+^ Calcd for C_23_H_33_N_3_O_4_Na 438.2369; Found 438.2368.

**Mp**. 147–149 °C.

(tert-butoxycarbonyl)-L-leucyl-L-alanine (26)

C_14_H_26_N_2_O_5_

**MW**: 302.37 g.mol^-1^

**Yield**: 85%

White solid

Following Procedure **A**, using *L*-Leucyl-*L*-alanine (500 mg, 2.47 mmol, 1 equiv.), Boc_2_O (539 mg, 2.47 mmol, 1 equiv.), Et_3_N (413 µL, 2.97 mmol, 1.2 equiv.) and MeOH (15 mL). The product was obtained as a white solid (634 mg, 85%).

**^1^H NMR (400 MHz, MeOD)** *δ* 4.39 (q, *J* = 7.2 Hz, 1H), 4.11 (dd, *J* = 9.9, 5.1 Hz, 1H), 1.78 – 1.65 (m, 1H), 1.61 – 1.47 (m, 2H), 1.44 (s, 9H), 1.40 (d, *J* = 7.3 Hz, 3H), 0.95 (t, *J* = 7.0 Hz, 6H), COOH and NH unobserved.

**^13^C NMR (101 MHz, MeOD)** *δ* 175.7, 175.4, 157.8, 80.6, 54.3, 42.2, 28.7, 25.8, 23.4, 21.9, 17.8.

**IR (cm^-1^)** 3324, 3297, 2950, 2930, 2869, 1680, 1639, 1530, 1448, 1291, 1250, 1170.

**HRMS** **(ESI-TOF) *m/z*** [M-H]^-^ Calcd for C_14_H_25_N_2_O_5_ 301.1764; Found 301.1765.

**Mp**. 145–147 °C.

Tert-butyl ((S)-4-methyl-1-oxo-1-(((S)-1-oxo-1-(prop-2-yn-1-ylamino)propan-2-yl)amino)pentan-2-yl)carbamate (27)

C_17_H_29_N_3_O_4_

**MW**: 339.44 g.mol^-1^

**Yield**: 86%

Yellow solid

Following Procedure **C**, using (*tert*-butoxycarbonyl)-*L*-leucyl-*L*-alanine **26** (200 mg, 0.661 mmol, 1 equiv.), propargylamine (50 µL, 0.794 mmol, 1.2 equiv.), HOBt (107 mg, 0.794 mmol, 1.2 equiv.), EDC hydrochloride (151 mg, 0.794 mmol, 1.2 equiv.), DIPEA (269 µL, 1.652 mmol, 2.5 equiv.) and DMF (8 mL). The crude product was purified by column chromatography (SiO_2_, DCM/MeOH, 95/05) to afford the desired product as a yellow solid (194 mg, 86%).

**^1^H NMR (400 MHz, MeOD)** *δ* 4.34 (dd, *J* = 14.0, 6.9 Hz, 1H), 4.07 (dd, *J* = 9.2, 5.5 Hz, 1H), 4.01 – 3.89 (m, 2H), 2.62 – 2.53 (m, 1H), 1.70 (dt, *J* = 13.3, 6.5 Hz, 1H), 1.57 – 1.49 (m, 2H), 1.45 (s, 9H), 1.34 (d, *J* = 7.1 Hz, 3H), 1.00 – 0.90 (m, 6H), NH unobserved.

**^13^C NMR (101 MHz, MeOD)** *δ* 175.5, 174.3, 158.1, 80.7, 80.3, 72.3, 54.6, 50.1, 41.9, 29.5, 28.7, 25.84, 23.5, 21.9, 18.1.

**IR (cm^-1^)** 3272, 2955, 1691, 1665, 1630, 1542, 1419, 1366, 1143.

**HRMS** **(ESI-TOF) *m/z*** [M+Na]^+^ Calcd for C_17_H_29_N_3_O_4_Na 362.2056; Found 362.2057.

**Mp**. 150–152 °C.

(tert-butoxycarbonyl)-L-tryptophylglycine (28)

C_18_H_23_N_3_O_5_

**MW**: 361.40 g.mol^-1^

**Yield**: 86%

White solid

Following Procedure **A**, using ***L*-Tryptophylglycine** (300 mg, 1.148 mmol, 1 equiv.), Boc_2_O (250 mg, 1.148 mmol, 1 equiv.), Et_3_N (192 µL, 1.378 mmol, 1.2 equiv.) and MeOH (15 mL). The product was obtained as a white solid (356 mg, 86%).

**^1^H NMR (400 MHz, MeOD)** *δ* 7.60 (d, *J* = 7.6 Hz, 1H), 7.32 (d, *J* = 8.0 Hz, 1H), 7.12 (s, 1H), 7.08 (t, *J* = 7.6 Hz, 1H), 7.01 (t, *J* = 7.4 Hz, 1H), 4.47 – 4.38 (m, 1H), 3.89 (s, 2H), 3.37 – 3.31 (m, 1H), 3.06 (dd, *J* = 14.5, 8.3 Hz, 1H), 1.39 – 1.19 (m, 9H), COOH and NH unobserved.

**^13^C NMR (101 MHz, MeOD)** δ 175.1, 172.8, 157.6, 138.0, 128.9, 124.6, 122.3, 119.7, 119.3, 112.2, 111.0, 80.7, 52.6, 41.9, 29.2, 28.6.

**IR (cm^-1^)** 3280, 2984, 2936, 1680, 1647, 1529, 1450, 1410, 1388, 1250, 1097, 967.

**HRMS** **(ESI-TOF) *m/z*** [M-H]^-^ Calcd for C_18_H_22_N_3_O_5_ 360.1559; Found 360.1562.

**Mp**. 139–141 °C.

Tert-butyl (S)-(3-(1H-indol-3-yl)-1-oxo-1-((2-oxo-2-(prop-2-yn-1-ylamino)ethyl)amino)propan-2-yl)carbamate (29)

C_21_H_26_N_4_O_4_

**MW**: 398.46 g.mol^-1^

**Yield**: 82%

Yellow solid

Following Procedure **C**, using (*tert*-butoxycarbonyl)-*L*-tryptophylglycine **28** (300 mg, 0.830 mmol, 1 equiv.), propargylamine (63 µL, 0.996 mmol, 1.2 equiv.), HOBt (134 mg, 0.996 mmol, 1.2 equiv.), EDC hydrochloride (190 mg, 0.996 mmol, 1.2 equiv.), DIPEA (361 µL, 2.075 mmol, 2.5 equiv.) and DMF (10 mL). The crude product was purified by column chromatography (SiO_2_, DCM/MeOH, 95/05) to afford the desired product as a yellow solid (272 mg, 82%).

**^1^H NMR (400 MHz, MeOD)** *δ* 7.57 (d, *J* = 7.7 Hz, 1H), 7.34 (d, *J* = 8.0 Hz, 1H), 7.11 (s, 1H), 7.12 – 7.06 (m, 1H), 7.02 (t, *J* = 7.3 Hz, 1H), 4.29 (t, *J* = 6.3 Hz, 1H), 3.94 (dd, *J* = 25.1, 7.6 Hz, 2H), 3.74 (dd, *J* = 69.2, 16.8 Hz, 2H), 3.17 (ddd, *J* = 62.8, 14.3, 6.9 Hz, 2H), 2.54 (s, 1H), 1.39 (s, 9H), NH unobserved.

**^13^C NMR (101 MHz, MeOD)** *δ* 175.5, 171.2, 158.0, 138.0, 128.7, 124.7, 122.4, 119.8, 119.3, 112.3, 110.8, 80.9, 80.3, 72.3, 57.5, 43.5, 29.4, 28.7, 28.6.

**IR (cm^-1^)** 3276, 2984, 2933, 1647, 1510, 1453, 1410, 1389, 1249, 1156, 1097, 951.

**HRMS** **(ESI-TOF) *m/z*** [M+Na]^+^ Calcd for C_21_H_26_N_4_O_4_Na 421.1852; Found 421.1855.

**Mp**. 92–94 °C.

**Synthesis of precursors 30-34**

**Scheme S5.** Synthesis of precursors **30-34**

Methyl L-leucylglycinate (30)

C_9_H_18_N_2_O_3_

**MW**: 202.25 g.mol^-1^

**Yield**: 99%

Yellow oil

To a solution of (*tert*-butoxycarbonyl)-*L*-leucylglycine (1 g, 3.47 mmol, 1 equiv.) in MeOH (10 mL) was added at 0 °C thionyl chloride (505 µL, 6.93 mmol, 2 equiv.) and the solution was stirred for 12 hours at room temperature. Then the solvent was removed under reduced pressure to aford the product as a yellow oil without further purification (700 mg, 99%).

**^1^H NMR (400 MHz, MeOD)** *δ* 4.03 (dd, *J* = 58.1, 17.5 Hz, 2H), 4.01 (t, *J* = 6.9 Hz, 1H), 3.73 (s, 3H), 1.93 – 1.64 (m, 3H), 1.02 (t, *J* = 5.6 Hz, 6H), NH unobserved.

**^13^C NMR (101 MHz, MeOD)** *δ* 171.21, 171.16, 52.9, 52.8, 41.8, 41.5, 25.2, 23.0, 22.4.

**IR (cm^-1^)** 2956, 2851, 1744, 1670, 1553, 1499, 1368, 1208, 1181, 1024, 981.

**HRMS** **(ESI-TOF) *m/z*** [M+H]^+^ Calcd for C_9_H_19_N_2_O_3_ 203.1396; Found 203.1394.

Prop-2-yn-1-yl-L-proline (31)

C_8_H_11_NO_2_

**MW**: 153.18 g.mol^-1^

**Yield**: 68%

Brown sticky solid

To a solution of *L*-proline (2.3 g, 20 mmol, 1 equiv.) in EtOH was added a solution of NaOH (1.6 g, 40 mmol, 2 equiv.) in water (5 mL). At 0 °C, propargyl bromide (solution 80% in toluene, 2.2 mL, 20 mmol, 1 equiv.) was added and the mixture was stirred at room temperature overnight. Then the reaction was acidified with HCl (2 M) until pH = 3-4. After evaporation of EtOH, AcOEt was added and the organic layer was extracted, dried over MgSO_4_ and the solvent was removed under reduced pressure to afford the product as a brown sticky solid without further purification (2.08 g, 68%).

**^1^H NMR (400 MHz, DMSO)** *δ* 3.99 – 3.79 (m, 2H), 3.77 – 3.65 (m, 1H), 3.52 (s, 1H), 3.33 – 3.24 (m, 1H), 2.91 (dd, *J* = 17.3, 8.9 Hz, 1H), 2.28 – 2.11 (m, 1H), 1.95 – 1.81 (m, 2H), 1.80 – 1.69 (m, 1H), COOH unobserved.

**^13^C NMR (101 MHz, DMSO)** *δ* 171.8, 78.4, 76.2, 64.3, 52.9, 41.7, 28.6, 22.5.

**HRMS** **(ESI-TOF) *m/z*** [M+H]^+^ Calcd for C_8_H_12_NO_2_ 154.0868; Found 154.0867.

The spectral data (^1^H-NMR) and (^13^C-NMR) was consistent with reported one.^[[2]](#footnote-2)^

Methyl prop-2-yn-1-yl-L-prolyl-L-leucylglycinate (32)

C_17_H_27_N_3_O_4_

**MW**: 337.42 g.mol^-1^

**Yield**: 87%

Colorless oil

Following Procedure **C**, using methyl *L*-leucylglycinate **30** (200 mg, 0.988 mmol, 1 equiv.), prop-2-yn-1-yl-*L*-proline **31** (151 mg, 0.988 mmol, 1 equiv.), HOBt (159 mg, 1.18 mmol, 1.2 equiv.), EDC hydrochloride (225 mg, 1.18 mmol, 1.2 equiv.), DIPEA (430 µL, 2.47 mmol, 2.5 equiv.) and DMF (10 mL). The crude product was purified by column chromatography (SiO_2_, DCM/MeOH, 95/05) to afford the desired product as a colorless oil (290 mg, 87%).

**^1^H NMR (400 MHz, MeOD)** *δ* 4.48 (t, *J* = 7.5 Hz, 1H), 4.40 (dd, *J* = 9.2, 6.6 Hz, 1H), 4.16 (qd, *J* = 16.5, 2.5 Hz, 2H), 3.94 (dd, *J* = 46.7, 17.6 Hz, 2H), 3.80 – 3.72 (m, 1H), 3.71 (s, 3H), 3.42 – 3.33 (m, 1H), 3.24 (t, *J* = 2.5 Hz, 1H), 2.64 – 2.52 (m, 1H), 2.26 – 2.12 (m, 2H), 2.12 – 2.01 (m, 1H), 1.81 – 1.68 (m, 1H), 1.67 – 1.59 (m, 2H), 0.96 (dd, *J* = 11.2, 6.4 Hz, 6H), NH unobserved.

**^13^C NMR (101 MHz, MeOD)** *δ* 174.7, 171.6, 168.8, 80.3, 73.2, 67.7, 55.7, 53.6, 52.7, 44.1, 41.7, 41.6, 30.9, 25.8, 24.1, 23.2, 21.9.

**IR (cm^-1^)** 3233, 2958, 2934, 1739, 1657, 1553, 1451, 1386, 1229, 1085, 1036, 944.

**HRMS** **(ESI-TOF) *m/z*** [M+H]^+^ Calcd for C_17_H_28_N_3_O_4_ 338.2080; Found 337.2082.

Prop-2-yn-1-yl-L-prolyl-L-leucylglycine (33)

C_16_H_25_N_3_O_4_

**MW**: 323.39 g.mol^-1^

**Yield**: 99%

Pale yellow oil

To a solution of **32** (333 mg, 0.987 mmol, 1 equiv.) in a mixture THF/MeOH (4 mL : 4 mL) was added a solution of NaOH (158 mg, 3.95 mmol, 4 equiv.) in H_2_O (4 mL). The mixture was stirred 1 hour at room temperature. The mixture was diluted with H_2_O and AcOEt and acidified with HCl (1M) until pH = 2. The organic layer was extracted, dried over MgSO_4_ and the solvent was removed under reduced pressure to afford the product as a pale yellow oil without further purification (318 mg, 99%).

**^1^H NMR (400 MHz, MeOD)** *δ* 4.52 – 4.44 (m, 2H), 4.23 (qd, *J* = 16.5, 2.5 Hz, 2H), 3.95 (dd, *J* = 46.0, 17.6 Hz, 2H), 3.85 – 3.75 (m, 1H), 3.49 – 3.34 (m, 1H), 3.30 (t, *J* = 2.3 Hz, 1H), 2.75 – 2.57 (m, 1H), 2.29 – 2.13 (m, 2H), 2.11 – 1.98 (m, 1H), 1.83 – 1.71 (m, 1H), 1.69 – 1.61 (m, 2H), 0.97 (dd, *J* = 12.0, 6.5 Hz, 6H), NH and COOH unobserved.

**^13^C NMR (101 MHz, MeOD)** *δ* 174.6, 171.5, 168.8, 80.3, 73.4, 67.8, 55.9, 53.6, 44.3, 41.7, 41.6, 31.0, 25.8, 24.1, 23.3, 22.0.

**IR (cm^-1^)** 3264, 2960, 2851, 1748, 1666, 1554, 1439, 1201, 1135, 799.

**HRMS** **(ESI-TOF) *m/z*** [M+H]^+^ Calcd for C_16_H_26_N_3_O_4_ 324.1923; Found 324.1924.

Tert-butyl(2-(2-((S)-4-methyl-2-((S)-1-(prop-2-yn-1-yl)pyrrolidine-2-carboxamido)pentanamido)acetamido)ethyl)carbamate (34)

C_23_H_39_N_5_O_5_

**MW**: 465.69 g.mol^-1^

**Yield**: 77%

Orange oil

Following Procedure **C**, using **33** (318 mg, 0.987 mmol, 1 equiv.), *tert*-butyl (2-aminoethyl)carbamate (189 mg, 1.18 mmol, 1.2 equiv.), HOBt (159 mg, 1.18 mmol, 1.2 equiv.), EDC hydrochloride (226 mg, 1.18 mmol, 1.2 equiv.), DIPEA (430 µL, 2.47 mmol, 2.5 equiv.) and DMF (10 mL). The crude product was purified by column chromatography (SiO_2_, DCM/MeOH, 95/05) to afford the desired product as an orange oil (318 mg, 77%).

**^1^H NMR (400 MHz, MeOD)** *δ* 4.39 (t, *J* = 7.0 Hz, 1H), 3.84 (dd, *J* = 66.5, 16.7 Hz, 2H), 3.59 (d, *J* = 1.2 Hz, 2H), 3.44 (dd, *J* = 9.9, 4.7 Hz, 1H), 3.27 (t, *J* = 5.4 Hz, 2H), 3.20 – 3.10 (m, 3H), 2.82 (td, *J* = 9.5, 6.6 Hz, 1H), 2.67 (t, *J* = 2.3 Hz, 1H), 2.28 – 2.14 (m, 1H), 1.93 – 1.74 (m, 3H), 1.68 – 1.61 (m, 3H), 1.43 (s, 9H), 0.96 (dd, *J* = 12.1, 6.2 Hz, 6H), NH unobserved.

**^13^C NMR (101 MHz, MeOD)** *δ* 176.7, 175.0, 171.5, 158.4, 80.1, 79.2, 74.9, 65.7, 53.6, 53.2, 43.5, 42.5, 41.7, 40.8, 40.7, 32.1, 28.8, 26.0, 25.1, 23.4, 22.1.

**IR (cm^-1^)** 3303, 2959, 2934, 1649, 1516, 1365, 1250, 1169.

**HRMS** **(ESI-TOF) *m/z*** [M+H]^+^ Calcd for C_23_H_40_N_5_O_5_ 466.3029; Found 466.3027.

**Synthesis of precursors 35-36**

**Scheme S6.** Synthesis of precursors **35-36**

Tert-butyl (4-(3-(4-aminophenoxy)phenoxy)phenyl)carbamate (35)

C_23_H_24_N_2_O_4_

**MW**: 392.45 g.mol^-1^

**Yield**: 99%

White solid

To a solution of 4,4'-(1,3-phenylenebis(oxy))dianiline (1 g, 3.42 mmol, 1 equiv.) in THF, was added Boc_2_O (746 mg, 3.42 mmol, 1 equiv.) and the solution was stirred overnight at 60 °C. Then the solvent was removed under reduced pressure. Purification on reverse phase (C18 column, 100% H_2_O with 0.1% TFA to 100% ACN with 0.1 %TFA) to afford the desired product as a white solid (1.34 g, 99%).

**^1^H NMR (400 MHz, MeOD)** *δ* 7.39 (d, *J* = 8.9 Hz, 2H), 7.35 – 7.28 (m, 3H), 7.13 – 7.08 (m, 2H), 6.98 – 6.92 (m, 2H), 6.71 (dddd, *J* = 8.2, 6.6, 2.3, 0.8 Hz, 2H), 6.55 (t, *J* = 2.3 Hz, 1H), 1.51 (s, 9H), NH unobserved.

**^13^C NMR (101 MHz, MeOD)** *δ* 161.1, 159.4, 157.9, 155.4, 152.7, 136.9, 131.9, 129.0, 124.9, 121.5, 121.13, 121.06, 114.1, 114.0, 109.5, 80.9, 28.7.

**IR (cm^-1^)** 2984, 2852, 1710, 1679, 1635, 1607, 1509, 1480, 1358, 1272, 1202, 1162, 1108.

**HRMS** **(ESI-TOF) *m/z*** [M+H]^+^ Calcd for C_23_H_25_N_2_O_4_ 393.1814; Found 393.1814.

**Mp**. 139–141 °C.

Tert-butyl (4-(3-(4-(pent-4-ynamido)phenoxy)phenoxy)phenyl)carbamate (36)

C_28_H_28_N_2_O_5_

**MW**: 472.54 g.mol^-1^

**Yield**: 71%

Yellow solid

Following Procedure **B**, using **35** (300 mg, 0.765 mmol, 1 equiv.), pent-4-ynoic acid (75 mg, 0.765 mmol, 1 equiv.), HATU (436 mg, 1.15 mmol, 1.5 equiv.), DIPEA (379 µL, 2.29 mmol, 3 equiv.) and DMF (8 mL). The crude product was purified by column chromatography (SiO_2_, DCM/MeOH, 98/02) to afford the desired product as a yellow solid (258 mg, 71%).

**^1^H NMR (400 MHz, MeOD)** *δ* 7.54 (d, *J* = 9.1 Hz, 2H), 7.38 (d, *J* = 8.9 Hz, 2H), 7.25 (t, *J* = 8.2 Hz, 1H), 7.00 – 6.90 (m, 4H), 6.64 (dd, *J* = 8.3, 2.3 Hz, 2H), 6.52 (t, *J* = 2.3 Hz, 1H), 2.60 – 2.52 (m, 4H), 2.29 (t, *J* = 2.5 Hz, 1H), 1.51 (s, 9H), NH unobserved.

**^13^C NMR (101 MHz, MeOD)** *δ* 172.2, 161.0, 160.6, 155.5, 154.2, 152.9, 136.7, 135.7, 131.6, 123.0, 121.0, 120.7, 113.2, 108.8, 83.5, 80.8, 70.3, 36.7, 28.7, 15.6, two carbons unobserved.

**IR (cm^-1^)** 3293, 2951, 2832, 1691, 1648, 1536, 1505, 1480, 1406, 1308, 1226, 1160, 1119, 1067, 956.

**HRMS** **(ESI-TOF) *m/z*** [M+Na]^+^ Calcd for C_28_H_28_N_2_O_5_Na 495.1896; Found 495.1895.

**Mp**. 108–110 °C.

**Preparation of substrates S1-13 for click reaction**

**Scheme S7.** Synthesis of substrates **S1-13.**

**General procedure D**

To a solution of Alkyne-*N*-Boc compound (1.5 equiv.) in DCM was added HCl (4M in 1.4-dioxane, 100 equiv.). The mixture was stirred overnight at room temperature. Then the solvent was removed under reduced pressure. The mixture was dissolved in DMF and added to a solution of sydnonimine **5** (1 equiv.), HATU (2 equiv.) and DIPEA (3 equiv.) in DMF which has been previously stirred for 20 minutes. The reaction was stirred for 5 hours at room temperature, and was concentrated. The crude product was purified by column chromatography with the indicated eluent to afford the desired product.

(3-(3-((2-oxo-2-(prop-2-yn-1-ylamino)ethyl)carbamoyl)phenyl)-1,2,3-oxadiazol-3-ium-5-yl)((pyren-1-ylmethyl)carbamoyl)amide (S1)

C_32_H_24_N_6_O_4_

**MW**: 556.58 g.mol^-1^

**Yield**: 36%

Orange solid

Following Procedure **D**, using **13** (343 mg, 1.62 mmol, 1.5 equiv.), HCl (4M in dioxane, 24 mL, 160 mmol, excess), DCM (3 mL). Then, **5** (498 mg, 1.08 mmol, 1 equiv.), HATU (820 mg, 2.16 mmol, 2 equiv.), DIPEA (536 µL, 3.24 mmol, 3 equiv.) and DMF (8 mL). The crude product was purified by column chromatography (SiO_2_, DCM/MeOH, 95/05) to afford the desired product as an orange solid (200 mg, 36%).

**^1^H NMR (400 MHz, DMSO-*d*6)** *δ* 9.13 (t, *J* = 5.9 Hz, 1H), 8.55 (s, 1H), 8.52 (d, *J* = 9.3 Hz, 1H), 8.50 – 8.49 (m, 1H), 8.49 – 8.45 (m, 1H), 8.31 (d, *J* = 3.7 Hz, 1H), 8.29 – 8.26 (m, 1H), 8.24 – 8.21 (m, 2H), 8.20 – 8.17 (m, 1H), 8.14 (s, 1H), 8.10 – 8.07 (m, 1H), 8.05 (d, *J* = 7.6 Hz, 1H), 7.84 (t, *J* = 6.4 Hz, 1H), 7.80 (t, *J* = 8.0 Hz, 1H), 5.03 (d, *J* = 6.1 Hz, 2H), 3.94 (d, *J* = 5.9 Hz, 2H), 3.90 (dd, *J* = 5.5, 2.5 Hz, 2H), 3.10 (t, *J* = 2.5 Hz, 1H)., NH unobserved.

**^13^C NMR (101 MHz, DMSO-*d*6)** *δ* 172.1, 168.4, 164.6, 160.8, 135.7, 134.4, 133.7, 131.5, 130.8, 130.4, 130.3, 129.8, 127.9, 127.4, 127.3, 126.7, 126.5, 126.10, 125.1, 125.0, 124.6, 124.6, 124.0, 124.0, 123.4, 120.8, 102.5, 79.1, 72.9, 42.5, 41.6, 27.9.

**IR (cm^-1^)** 3288, 2654, 2832, 1633, 1584, 1503, 1448, 1349, 1263, 1212, 1024, 964.

**HRMS** **(ESI-TOF) *m/z*** [M-H]^-^ Calcd for C_32_H_23_N_6_O_4_ 555.1781; Found 555.1784.

**Mp**. 128–130 °C.

(3-(3-((5-oxo-5-(prop-2-yn-1-ylamino)pentyl)carbamoyl)phenyl)-1,2,3-oxadiazol-3-ium-5-yl)((pyren-1-ylmethyl)carbamoyl)amide (S2)

C_35_H_30_N_6_O_4_

**MW**: 598.66 g.mol^-1^

**Yield**: 54%

Orange solid

Following Procedure **D**, using **14** (96 mg, 0.324 mmol, 1.5 equiv.), HCl (4M in dioxane, 7.6 mL, 32.4 mmol, excess), DCM (3 mL). Then, **5** (100 mg, 0.216 mmol, 1 equiv.), HATU (164 mg, 0.432 mmol, 2 equiv.), DIPEA (103 µL, 0.648 mmol, 3 equiv.) and DMF (5 mL). The crude product was purified by column chromatography (SiO_2_, DCM/MeOD, 95/05) to afford the desired product as an orange solid (69 mg, 54%).

**^1^H NMR (400 MHz, DMSO-*d*6**) *δ* 8.77 (t, *J* = 5.3 Hz, 1H), 8.56 (s, 1H), 8.53 (d, *J* = 9.3 Hz, 1H), 8.43 (s, 1H), 8.35 – 8.25 (m, 3H), 8.18 (d, *J* = 7.5 Hz, 2H), 8.15 (s, 1H), 8.11 – 8.04 (m, 2H), 7.87 – 7.81 (m, 1H), 7.78 (t, *J* = 8.0 Hz, 1H), 5.04 (d, *J* = 5.6 Hz, 2H), 3.85 (dd, *J* = 5.3, 2.4 Hz, 2H), 3.36 – 3.26 (m, 2H), 3.06 (t, *J* = 2.4 Hz, 1H), 2.14 (t, *J* = 6.6 Hz, 2H), 1.64 – 1.46 (m, 4H), NH unobserved.

**^13^C NMR (101 MHz, DMSO-*d*6)** *δ* 171.9, 171.7, 164.0, 160.6, 136.2, 134.3, 133.7, 131.3, 130.8, 130.4, 130.3, 129.8, 127.9, 127.4, 127.3, 126.7, 126.5, 126.1, 125.0, 125.0, 124.6, 124.4, 123.99, 123.97, 123.4, 120.6, 102.6, 81.3, 72.7, 41.5, 34.7, 28.6, 27.7, 22.7, one carbon hidden.

**IR (cm^-1^)** 3300, 3040, 2940, 2917, 2126, 1628, 1528, 1442, 1281, 1211, 1103, 840.

**HRMS** **(ESI-TOF) *m/z*** [M+H]^+^ Calcd for C_35_H_31_N_6_O_4_ 599.2407; Found 599.2408.

**Mp**. 158–160 °C.

(3-(3-((8-oxo-8-(prop-2-yn-1-ylamino)octyl)carbamoyl)phenyl)-1,2,3-oxadiazol-3-ium-5-yl)((pyren-1-ylmethyl)carbamoyl)amide (S3)

C_38_H_36_N_6_O_4_

**MW**: 640.74 g.mol^-1^

**Yield**: 42%

Orange solid

Following Procedure **D**, using **15** (232 mg, 0.784 mmol, 1.5 equiv.), HCl (4M in dioxane, 16 mL, 68 mmol, excess), DCM (3 mL). Then, **5** (241 mg, 0.523 mmol, 1 equiv.), HATU (397 mg, 1.046 mmol, 2 equiv.), DIPEA (249 µL, 1.569 mmol, 3 equiv.) and DMF (8 mL). The crude product was purified by column chromatography (SiO_2_, DCM/MeOD, 95/05) to afford the desired product as an orange solid (140 mg, 42%).

**^1^H NMR (400 MHz, DMSO-*d*6)** *δ* 8.77 (t, *J* = 5.5 Hz, 1H), 8.54 (s, *J* = 10.5 Hz, 1H), 8.53 (d, *J* = 10.5 Hz, 1H), 8.43 – 8.41 (m, 1H), 8.33 – 8.27 (m, 2H), 8.26 – 8.21 (m, 2H), 8.20 – 8.14 (m, 2H), 8.11 – 8.05 (m, 2H), 7.84 (t, *J* = 6.1 Hz, 1H), 7.78 (t, *J* = 8.0 Hz, 1H), 5.03 (d, *J* = 6.1 Hz, 2H), 3.83 (dd, *J* = 5.5, 2.5 Hz, 2H), 3.29 (dd, *J* = 12.9, 6.6 Hz, 2H), 3.07 (t, *J* = 2.5 Hz, 1H), 2.07 (t, *J* = 7.4 Hz, 2H), 1.59 – 1.43 (m, 4H), 1.29 – 1.21 (m, 6H), NH unobserved.

**^13^C NMR (101 MHz, DMSO-*d*6)** *δ* 172.1, 171.9, 164.1, 160.9, 136.3, 134.4, 133.8, 131.3, 130.8, 130.4, 130.3, 129.8, 127.9, 127.4, 127.3, 126.8, 126.5, 126.2, 125.1, 125.0, 124.6, 124.4, 124.0, 124.0, 123.5, 120.6, 102.6, 81.4, 72.8, 53.6, 41.8, 35.0, 29.0, 28.6, 25.1, 18.1, 16.7, 12.5.

**IR (cm^-1^)** 3296, 2937, 2917,2845, 1664, 1631, 1528, 1389, 1281, 1111, 842.

**HRMS** **(ESI-TOF) *m/z*** [M+H]^+^ Calcd for C_38_H_37_N_6_O_4_ 641.2877; Found 641.2877.

**Mp**. 161–163 °C.

(3-(3-((11-oxo-11-(prop-2-yn-1-ylamino)undecyl)carbamoyl)phenyl)-1,2,3-oxadiazol-3-ium-5-yl)((pyren-1-ylmethyl)carbamoyl)amide (S4)

C_41_H_42_N_2_O_4_

**MW**: 682.82 g.mol^-1^

**Yield**: 48%

Orange solid

Following Procedure **D**, using **16** (170 mg, 0.503 mmol, 1.5 equiv.), HCl (4M in dioxane, 11.9 mL, 50 mmol, excess), DCM (3 mL). Then, **5** (154 mg, 0.335 mmol, 1 equiv.), HATU (254 mg, 0.670 mmol, 2 equiv.), DIPEA (160 µL, 1.005 mmol, 3 equiv.) and DMF (8 mL). The crude product was purified by column chromatography (SiO_2_, DCM/MeOD, 95/05) to afford the desired product as an orange solid (109 mg, 48%).

**^1^H NMR (400 MHz, DMSO-*d*6)** *δ* 8.74 (t, *J* = 5.5 Hz, 1H), 8.54 (s, 1H), 8.41 (t, *J* = 1.7 Hz, 1H), 8.29 (t, *J* = 7.3 Hz, 2H), 8.24 (t, *J* = 8.7 Hz, 2H), 8.20 – 8.14 (m, 3H), 8.10 – 8.04 (m, 2H), 7.85 – 7.74 (m, 2H), 5.03 (d, *J* = 6.1 Hz, 2H), 3.82 (dd, *J* = 5.5, 2.5 Hz, 2H), 3.66 – 3.56 (m, 1H), 3.35 – 3.25 (m, 2H), 3.19 – 3.09 (m, 1H), 3.05 (t, *J* = 2.5 Hz, 1H), 2.05 (t, *J* = 7.4 Hz, 2H), 1.56 – 1.51 (m, 2H), 1.50 – 1.37 (m, 2H), 1.30 – 1.17 (m, 12H), NH unobserved.

**^13^C NMR (101 MHz, DMSO-*d*6)** *δ* 172.0, 171.8, 164.0, 160.8, 136.3, 134.4, 133.8, 131.3, 130.8, 130.4, 130.3, 129.8, 127.9, 127.4, 127.3, 126.8, 126.5, 126.1, 125.1, 125.0, 124.6, 124.4, 124.0, 123.4, 120.57, 102.6, 81.3, 72.7, 53.6, 41.8, 35.0, 29.0, 28.9, 28.7, 28.6, 27.7, 26.4, 25.1, 18.1, 16.7, 12.4.

**IR (cm^-1^)** 3294, 2920, 2850, 1642, 1630, 1537, 1466, 1300.

**HRMS** **(ESI-TOF) *m/z*** [M+H]^+^ Calcd for C_41_H_43_N_6_O_4_ 683.3346; Found 683.3345.

**Mp**. 164–166 °C.

(3-(3-((11-oxo-11-((2-oxo-2-(prop-2-yn-1-ylamino)ethyl)amino)undecyl)carbamoyl)phenyl)-1,2,3-oxadiazol-3-ium-5-yl)((pyren-1-ylmethyl)carbamoyl)amide (S5)

C_43_H_45_N_7_O_5_

**MW**: 739.88 g.mol^-1^

**Yield**: 65%

Orange solid

Following Procedure **D**, using **22** (430 mg, 1.09 mmol, 1.5 equiv.), HCl (4M in dioxane, 15 mL, 64 mmol, excess), DCM (3 mL). Then, **5** (335 mg, 0.727 mmol, 1 equiv.), HATU (551 mg, 1.45 mmol, 2 equiv.), DIPEA (360 µL, 2.18 mmol, 3 equiv.) and DMF (10 mL). The crude product was purified by column chromatography (SiO_2_, DCM/MeOD, 95/05) to afford the desired product as an orange solid (349 mg, 65%).

**^1^H NMR (400 MHz, DMSO-*d*6)** *δ* 8.76 (t, *J* = 5.5 Hz, 1H), 8.54 (s, 1H), 8.42 (t, *J* = 1.8 Hz, 1H), 8.33 – 8.23 (m, 3H), 8.17 (dd, *J* = 8.0, 1.9 Hz, 2H), 8.15 (s, 1H), 8.08 (d, *J* = 7.8 Hz, 1H), 8.08 (t, *J* = 7.6 Hz, 1H), 8.01 (t, *J* = 5.7 Hz, 1H), 7.84 (t, *J* = 6.1 Hz, 1H), 7.79 (t, *J* = 8.0 Hz, 1H), 5.03 (d, *J* = 6.2 Hz, 2H), 3.85 (dd, *J* = 5.6, 2.5 Hz, 2H), 3.65 (d, *J* = 5.9 Hz, 2H), 3.17 (d, *J* = 5.2 Hz, 2H), 3.10 (t, *J* = 2.5 Hz, 1H), 2.11 (t, *J* = 7.5 Hz, 2H), 1.61 – 1.41 (m, 4H), 1.34 – 1.15 (m, 12H), NH unobserved.

**^13^C NMR (101 MHz, DMSO-*d*6)** *δ* 171.5, 171.4, 171.3, 163.5, 160.2, 135.7, 133.8, 133.2, 130.7, 130.3, 129.8, 129.8, 129.3, 127.4, 126.9, 126.7, 126.2, 126.0, 125.6, 124.5, 124.5, 124.1, 123.8, 123.5, 122.9, 120.0, 102.1, 80.8, 72.1, 53.1, 41.3, 41.0, 34.5, 28.5, 28.4, 28.2, 28.0, 27.1, 25.9, 24.6, 17.5, 16.2, 11.9.

**IR (cm^-1^)** 3296, 2956, 2934, 2886, 1670, 1642, 1630, 1537, 1466, 1250.

**HRMS** **(ESI-TOF) *m/z*** [M+H]^+^ Calcd for C_43_H_46_N_7_O_5_ 740.3560; Found 740.3558.

**Mp**. 163–165 °C.

(3-(3-((11-oxo-11-((5-oxo-5-(prop-2-yn-1-ylamino)pentyl)amino)undecyl)carbamoyl)phenyl)-1,2,3-oxadiazol-3-ium-5-yl)((pyren-1-ylmethyl)carbamoyl)amide (S6)

C_46_H_51_N_7_O_5_

**MW**: 781.96 g.mol^-1^

**Yield**: 59%

Orange solid

Following Procedure **D**, using **23** (226 mg, 0.516 mmol, 1.5 equiv.), HCl (4M in dioxane, 11 mL, 32.4 mmol, excess), DCM (3 mL). Then, **5** (159 mg, 0.344 mmol, 1 equiv.), HATU (261 mg, 0.688 mmol, 2 equiv.), DIPEA (172 µL, 1.032 mmol, 3 equiv.) and DMF (8 mL). The crude product was purified by column chromatography (SiO_2_, DCM/MeOD, 95/05) to afford the desired product as an orange solid (158 mg, 59%).

**^1^H NMR (400 MHz, DMSO-*d*6)** *δ* 8.75 (t, *J* = 5.5 Hz, 1H), 8.56 – 8.50 (m, 1H), 8.43 – 8.40 (m, 1H), 8.29 (t, *J* = 7.3 Hz, 2H), 8.25 (t, *J* = 8.6 Hz, 2H), 8.19 – 8.14 (m, 3H), 8.07 (t, *J* = 7.7 Hz, 1H), 7.86 – 7.75 (m, 2H), 7.73 – 7.67 (m, 1H), 5.03 (d, *J* = 6.1 Hz, 2H), 3.82 (dd, *J* = 5.5, 2.5 Hz, 2H), 3.06 (t, *J* = 2.5 Hz, 1H), 2.99 (dd, *J* = 12.6, 6.7 Hz, 2H), 2.06 (t, *J* = 7.4 Hz, 2H), 2.01 (t, *J* = 7.4 Hz, 2H), 1.58 – 1.50 (m, 2H), 1.49 – 1.41 (m, 4H), 1.36 – 1.27 (m, 4H), 1.26 – 1.18 (m, 12H), NH unobserved.

**^13^C NMR (101 MHz, DMSO-*d*6)** *δ* 172.1, 171.9, 171.7, 164.1, 160.9, 136.3, 134.4, 133.8, 133.7, 131.3, 130.8, 130.4, 130.3, 129.8, 127.9, 127.4, 127.3, 126.8, 126.5, 126.1, 125.1, 125.0, 124.6, 124.4, 124.0, 123.4, 120.6, 102.5, 81.3, 72.7, 46.3, 41.5, 38.1, 35.4, 34.7, 29.0, 28.9, 28.9, 28.8, 28.7, 28.6, 27.7, 26.5, 25.3, 22.6, 18.8.

**IR (cm^-1^)** 3296, 2960, 2928, 2871, 1685, 1637, 1526, 1448, 1389, 1249.

**HRMS** **(ESI-TOF) *m/z*** [M+H]^+^ Calcd for C_46_H_52_N_7_O_5_ 782.4030; Found 782.4034.

**Mp**. 168–170 °C.

(3-(3-((2-(2-(2-(prop-2-yn-1-yloxy)ethoxy)ethoxy)ethyl)carbamoyl)phenyl)-1,2,3-oxadiazol-3-ium-5-yl)((pyren-1-ylmethyl)carbamoyl)amide (S7)

C_36_H_33_N_5_O_6_

**MW**: 631.69 g.mol^-1^

**Yield**: 71%

Sticky solid

To a solution of sydnonimine **5** (205mg, 0.444 mmol, 1 equiv.) in DMF (5 mL) were added HATU (337 mg, 0.887 mmol, 2 equiv.) and DIPEA (220 µL, 1.322 mmol, 3 equiv.), the solution was stirred for 20 minutes. Then 2-(2-(2-(prop-2-yn-1-yloxy)ethoxy)ethoxy)ethan-1-amine (83 mg, 0.444 mmol, 1 equiv.) was added and the mixture was stirred for 5 hours at room temperature. The solvent was removed under reduced pressure and the crude product was purified by column chromatography (SiO_2_, DCM/MeOD, 95/05) to afford the desired product as a sticky solid (200 mg, 71%).

**^1^H NMR (400 MHz, DMSO-*d*6)** *δ* 8.89 (t, *J* = 5.4 Hz, 1H), 8.55 (s, 1H), 8.52 (d, *J* = 9.3 Hz, 1H), 8.44 (s, 1H), 8.32 – 8.20 (m, 4H), 8.19 – 8.15 (m, 2H), 8.14 (s, 2H), 8.09 (d, *J* = 6.0 Hz, 1H), 8.06 (t, *J* = 6.7 Hz, 1H), 7.85 (t, *J* = 6.1 Hz, 1H), 7.78 (t, *J* = 8.0 Hz, 1H), 5.03 (d, *J* = 5.9 Hz, 2H), 4.10 (d, *J* = 2.4 Hz, 2H), 3.61 – 3.44 (m, 12H), 3.40 (t, *J* = 2.4 Hz, 1H).

**^13^C NMR (101 MHz, DMSO-*d*6)** *δ* 172.1, 164.3, 160.9, 136.0, 134.4, 133.8, 131.4, 130.8, 130.4, 130.3, 129.8, 127.9, 127.4, 127.3, 126.8, 126.5, 126.1, 125.1, 125.0, 124.6, 124.5, 124.0, 124.0, 123.4, 120.6, 102.5, 80.3, 77.1, 69.7, 69.7, 69.5, 68.8, 68.5, 57.5, 53.6, 41.6.

**IR (cm^-1^)** 3204,2956, 2834, 1681, 1627, 1503, 1408, 1308, 1250, 1211, 1122, 999.

**HRMS** **(ESI-TOF) *m/z*** [M+H]^+^ Calcd for C_36_H_34_N_5_O_6_ 632.2509; Found 632.2512.

(3-(3-((4-(3-(4-(pent-4-ynamido)phenoxy)phenoxy)phenyl)carbamoyl)phenyl)-1,2,3-oxadiazol-3-ium-5-yl)((pyren-1-ylmethyl)carbamoyl)amide (S8)

C_50_H_36_N_6_O_6_

**MW**: 816.87 g.mol^-1^

**Yield**: 69%

Beige solid

Following Procedure **D**, using **36** (184 mg, 0.390 mmol, 1.5 equiv.), HCl (4M in dioxane, 9.1 mL, 39.0 mmol, excess), DCM (4 mL). Then, **5** (120 mg, 0.260 mmol, 1 equiv.), HATU (197 mg, 0.520 mmol, 2 equiv.), DIPEA (129 µL, 0.780 mmol, 3 equiv.) and DMF (5 mL). The crude product was purified by column chromatography (SiO_2_, DCM/MeOD, 95/05) to afford the desired product as a beige solid (146 mg, 69%).

**^1^H NMR (400 MHz, DMSO-*d*6)** *δ* 10.55 (s, 1H), 10.02 (s, 1H), 8.59 (s, 1H), 8.58 – 8.55 (m, 1H), 8.53 (d, *J* = 9.3 Hz, 1H), 8.29 (t, *J* = 8.8 Hz, 3H), 8.24 (d, *J* = 9.2 Hz, 2H), 8.15 (s, 2H), 8.07 (t, *J* = 8.2 Hz, 2H), 7.89 – 7.82 (m, 2H), 7.79 (d, *J* = 9.0 Hz, 2H), 7.62 (d, *J* = 9.0 Hz, 2H), 7.33 (t, *J* = 8.2 Hz, 1H), 7.10 (d, *J* = 9.0 Hz, 2H), 7.06 – 7.01 (m, 2H), 6.68 (td, *J* = 7.9, 2.2 Hz, 2H), 6.57 (t, *J* = 2.3 Hz, 1H), 5.03 (d, *J* = 6.1 Hz, 2H), 3.67 – 3.50 (m, 1H), 3.17 – 3.06 (m, 2H), 2.80 (t, *J* = 2.5 Hz, 1H), 2.47 – 2.40 (m, 2H).

**^13^C NMR (101 MHz, DMSO-*d*6)** *δ* 172.1, 169.2, 166.6, 163.4, 160.9, 158.9, 158.6, 152.0, 151.0, 136.5, 135.4, 134.8, 134.4, 133.8, 131.7, 131.0, 130.8, 130.5, 130.3, 129.8, 127.9, 127.4, 127.3, 126.8, 126.5, 126.2, 125.1, 125.0, 124.6, 124.0, 124.0, 123.4, 122.3, 121.3, 120.7, 119.9, 119.6, 112.1, 112.0, 107.4, 102.8, 83.6, 71.5, 53.6, 35.1, 14.1.

**IR (cm^-1^)** 3204, 1627, 1503, 1408, 1308, 1211, 1122, 966, 835.

**HRMS** **(ESI-TOF) *m/z*** [M+H]^+^ Calcd for C_50_H_37_N_6_O_6_ 817.2775; Found 817.2780.

**Mp**. 152–154 °C.

(S)-(3-(3-((5-(hex-5-ynamido)-6-methoxy-6-oxohexyl)carbamoyl)phenyl)-1,2,3-oxadiazol-3-ium-5-yl)((pyren-1-ylmethyl)carbamoyl)amide (S9)

C_40_H_38_N_6_O_6_

**MW**: 698.78 g.mol^-1^

**Yield**: 64%

Yellow solid

Following Procedure **D**, using **6** (115 mg, 0.324 mmol, 1.5 equiv.), HCl (4M in dioxane, 7.6 mL, 32.4 mmol, excess), DCM (3 mL). Then, **5** (100 mg, 0.216 mmol, 1 equiv.), HATU (164 mg, 0.432 mmol, 2 equiv.), DIPEA (103 µL, 0.648 mmol, 3 equiv.) and DMF (5 mL). The crude product was purified by column chromatography (SiO_2_, DCM/MeOD, 95/05) to afford the desired product as an orange solid (96 mg, 64%).

**^1^H NMR (400 MHz, CDCl_3_)** *δ* 8.27 (s, 1H), 8.26-8.20 (m, 2H), 8.14 (d, *J* = 7.6 Hz, 2H), 8.11-8.03 (m, 3H), 8.02-7.95 (m, 4H), 7.69 (dd, *J* = 8.1, 1.2 Hz, 1H), 7.49 (t, *J* = 8.0 Hz, 1H), 6.38 (d, *J* = 7.9 Hz, 1H), 6.17 (t, *J* = 5.7 Hz, 1H), 5.12 (d, *J* = 5.7 Hz, 2H), 4.59 (td, *J* = 8.6, 4.4 Hz, 1H), 3.71 (s, 3H), 3.43 (dd, *J* = 12.1, 6.3 Hz, 2H), 2.38-2.26 (m, 2H), 2.16 (td, *J* = 6.9, 2.8 Hz, 2H), 1.93 (t, *J* = 2.6 Hz, 1H), 1.89-1.77 (m, 3H), 1.76-1.64 (m, 4H), 1.47-1.35 (m, 2H).

**^13^C NMR (101 MHz, CDCl_3_)** *δ* 173.1, 172.8, 172.7, 165.1, 161.7, 137.1, 136.8, 134.2, 131.6, 131.5, 131.0, 130.7, 129.9, 128.7, 127.6, 127.4, 127.3, 126.7, 125.9, 125.2, 125.1, 124.9 (2C), 124.8, 123.8, 123.6, 120.4, 102.1, 83.3, 69.5, 52.7, 51.6, 40.5, 35.1, 32.8, 30.4, 28.2, 24.2, 22.7, 17.8.

**IR (cm^-1^)** 3298, 2954, 2859, 1680, 1660, 1567, 1466, 1291,1250, 1206, 1001.

**HRMS** **(ESI-TOF) *m/z*** [M+H]^+^ Calcd for C_40_H_39_N_6_O_5_ 699.2930; Found 699.2931.

**Mp**. 149–151 °C.

(3-(3-(((S)-4-methyl-1-oxo-1-(((S)-1-oxo-1-(prop-2-yn-1-ylamino)propan-2-yl)amino)pentan-2-yl)carbamoyl)phenyl)-1,2,3-oxadiazol-3-ium-5-yl)((pyren-1-ylmethyl)carbamoyl)amide (S10)

C_39_H_37_N_7_O_5_

**MW**: 683.77 g.mol^-1^

**Yield**: 69%

Orange solid

Following Procedure **D**, using **27** (109 mg, 0.324 mmol, 1.5 equiv.), HCl (4M in dioxane, 7.6 mL, 32.4 mmol, excess), DCM (3 mL). Then, **5** (100 mg, 0.216 mmol, 1 equiv.), HATU (164 mg, 0.432 mmol, 2 equiv.), DIPEA (103 µL, 0.648 mmol, 3 equiv.) and DMF (5 mL). The crude product was purified by column chromatography (SiO_2_, DCM/MeOD, 95/05) to afford the desired product as an orange solid (101 mg, 69%).

**^1^H NMR (400 MHz, CDCl_3_/MeOD 1:1)** *δ* 8.10 (d, *J* = 1.5 Hz, 1H), 8.04 – 7.97 (m, 2H), 7.91 – 7.79 (m, 5H), 7.75 – 7.70 (m, 3H), 7.69 – 7.61 (m, 2H), 7.39 (t, *J* = 7.3 Hz, 1H), 7.12 (d, *J* = 1.8 Hz, 1H), 4.85 (s, 2H), 4.41 – 4.29 (m, 1H), 4.13 – 4.02 (m, 1H), 3.66 (d, *J* = 2.0 Hz, 2H), 3.05 – 3.00 (m, 1H), 2.01 – 1.96 (m, 1H), 1.47 – 1.35 (m, 2H), 1.11 – 1.00 (m, 5H), 0.66 (t, *J* = 5.2 Hz, 6H), NH unobserved.

**^13^C NMR (101 MHz, CDCl_3_/MeOD 1 :1)** *δ* 172.9, 172.6, 172.4, 165.5, 161.2, 135.9, 133.8, 131.8, 131.1, 130.7, 130.6, 130.48, 128.5, 127.7, 127.2, 127.0, 125.9, 125.8, 125.0, 125.0, 124.7, 124.6, 124.5, 124.1, 122.5, 120.6, 102.4, 78.9, 71.2, 54.5, 52.6, 42.4, 40.5, 28.7, 24.7, 22.6, 21.3, 17.3, one carbon hidden.

**IR (cm^-1^)** 3278, 2957, 2871, 1632, 1531, 1446, 1276, 1220, 1155.

**HRMS** **(ESI-TOF) *m/z*** [M+H]^+^ Calcd for C_39_H_38_N_7_O_5_ 684.2935; Found 684.2939.

**Mp**. 135–137 °C.

(3-(3-(((S)-1-(((S)-4-methyl-1-oxo-1-(prop-2-yn-1-ylamino)pentan-2-yl)amino)-1-oxo-3-phenylpropan-2-yl)carbamoyl)phenyl)-1,2,3-oxadiazol-3-ium-5-yl)((pyren-1-ylmethyl)carbamoyl)amide (S11)

C_45_H_41_N_7_O_5_

**MW**: 759.87 g.mol^-1^

**Yield**: 66%

Orange solid

Following Procedure **D**, using **25** (134 mg, 0.324 mmol, 1.5 equiv.), HCl (4M in dioxane, 7.6 mL, 32.4 mmol, excess), DCM (3 mL). Then, **5** (100 mg, 0.216 mmol, 1 equiv.), HATU (164 mg, 0.432 mmol, 2 equiv.), DIPEA (103 µL, 0.648 mmol, 3 equiv.) and DMF (5 mL). The crude product was purified by column chromatography (SiO_2_, DCM/MeOD, 95/05) to afford the desired product as an orange solid (108 mg, 66%).

**^1^H NMR (400 MHz, DMSO-*d*6)** *δ* 8.92 (d, *J* = 8.4 Hz, 1H), 8.56 – 8.48 (m, 2H), 8.36 – 8.33 (m, 1H), 8.32 – 8.29 (m, 2H), 8.27 (d, *J* = 3.1 Hz, 1H), 8.23 (d, *J* = 9.7 Hz, 2H), 8.18 – 8.15 (m, 1H), 8.14 (s, 1H), 8.12 – 8.09 (m, 1H), 8.08 – 8.04 (m, 1H), 7.82 (t, *J* = 6.1 Hz, 1H), 7.76 (t, *J* = 8.0 Hz, 1H), 7.36 – 7.33 (m, 2H), 7.27 – 7.23 (m, 2H), 7.18 – 7.13 (m, 1H), 5.03 (d, *J* = 6.1 Hz, 2H), 4.87 – 4.76 (m, 1H), 4.37 – 4.25 (m, 1H), 3.85 (dd, *J* = 5.4, 2.5 Hz, 2H), 3.65 – 3.56 (m, 1H), 3.22 – 3.12 (m, 2H), 3.10 (t, *J* = 2.0 Hz, 1H), 2.96 (dd, *J* = 13.8, 10.8 Hz, 1H), 1.65 – 1.55 (m, 1H), 1.54 – 1.40 (m, 2H), 0.85 (dd, *J* = 18.5, 6.5 Hz, 6H), one NH unobserved.

**^13^C NMR (101 MHz, DMSO-*d*6)** *δ* 172.1, 171.6, 170.9, 164.3, 160.8, 138.1, 135.7, 134.4, 133.7, 131.4, 130.8, 130.4, 130.3, 129.8, 129.1, 128.1, 128.0, 127.9, 127.4, 127.3, 126.8, 126.5, 126.2, 126.1, 125.1, 125.0, 124.6, 124.0, 124.0, 123.4, 120.8, 102.6, 81.0, 73.0, 54.7, 53.6, 51.0, 27.9, 24.1, 22.9, 21.6, 18.1, 16.7.

**IR (cm^-1^)** 3296, 2959, 2932, 2871, 1685, 1637, 1527, 1448, 1318, 1170.

**HRMS** **(ESI-TOF) *m/z*** [M+H]^+^ Calcd for C_45_H_42_N_7_O_5_ 760.3248; Found 760.3250.

**Mp**. 153–155 °C.

(S)-(3-(3-((3-(1H-indol-3-yl)-1-oxo-1-((2-oxo-2-(prop-2-yn-1-ylamino)ethyl)amino)propan-2-yl)carbamoyl)phenyl)-1,2,3-oxadiazol-3-ium-5-yl)((pyren-1-ylmethyl)carbamoyl)amide (S12)

C_43_H_34_N_8_O_5_

**MW**: 742.80 g.mol^-1^

**Yield**: 67%

Orange solid

Following Procedure **D**, using **29** (394 mg, 0.990 mmol, 1.5 equiv.), HCl (4M in dioxane, 15 mL, 99 mmol, excess), DCM (5 mL). Then, **5** (304 mg, 0.660 mmol, 1 equiv.), HATU (501 mg, 1.32 mmol, 2 equiv.), DIPEA (327 µL, 1.98 mmol, 3 equiv.) and DMF (8 mL). The crude product was purified by column chromatography (SiO_2_, DCM/MeOD, 95/05) to afford the desired product as an orange solid (258 mg, 67%).

**^1^H NMR (400 MHz, DMSO-*d*6)** *δ* 10.77 (s, 1H), 9.00 (d, *J* = 7.8 Hz, 1H), 8.58 – 8.47 (m, 2H), 8.41 (s, 1H), 8.30 (t, *J* = 6.2 Hz, 1H), 8.25 (t, *J* = 9.1 Hz, 1H), 8.21 – 8.16 (m, 2H), 8.14 (d, *J* = 9.1 Hz, 2H), 8.07 (t, *J* = 7.7 Hz, 2H), 7.85 (t, *J* = 5.9 Hz, 1H), 7.76 (t, *J* = 8.0 Hz, 1H), 7.68 (d, *J* = 7.7 Hz, 1H), 7.29 (d, *J* = 8.0 Hz, 1H), 7.21 (s, 1H), 7.03 (t, *J* = 7.5 Hz, 1H), 6.96 (t, *J* = 7.4 Hz, 1H), 5.04 (d, *J* = 5.8 Hz, 2H), 4.85 – 4.77 (m, 1H), 3.91 – 3.86 (m, 2H), 3.74 (qd, *J* = 16.6, 5.7 Hz, 2H), 3.31 – 3.13 (m, 2H), 3.11 (t, *J* = 2.5 Hz, 1H).

**^13^C NMR (101 MHz, DMSO-*d*6)** *δ* 172.1, 171.7, 168.5, 164.5, 160.9, 136.0, 135.6, 134.4, 133.6, 131.6, 130.8, 130.3, 129.8, 127.9, 127.4, 127.3, 127.3, 126.8, 126.5, 126.1, 125.1, 125.0, 124.7, 124.6, 124.0, 124.0, 123.6, 123.4, 121.0, 120.9, 120.8, 118.4, 118.2, 111.3, 110.3, 102.6, 80.9, 73.1, 54.9, 54.5, 42.0, 41.6, 27.9.

**IR (cm^-1^)** 3282, 2922, 2834, 1732, 1670, 1633, 1515, 1438, 1395, 1267, 1212, 1105, 962.

**HRMS** **(ESI-TOF) *m/z*** [M+H]^+^ Calcd for C_43_H_35_N_8_O_5_ 743.2731; Found 743.2728.

**Mp**. 151–153 °C.

(3-(3-((2-(2-((S)-4-methyl-2-((S)-1-(prop-2-yn-1-yl)pyrrolidine-2-carboxamido)pentanamido)acetamido)ethyl)carbamoyl)phenyl)-1,2,3-oxadiazol-3-ium-5-yl)((pyren-1-ylmethyl)carbamoyl)amide (S13)

C_45_H_47_N_9_O_6_

**MW**: 809.93 g.mol^-1^

**Yield**: 76%

Yellow solid

Following Procedure **D**, using **34** (180 mg, 0.387 mmol, 1.5 equiv.), HCl (4M in dioxane, 6 mL, 38.7 mmol, excess), DCM (4 mL). Then, **5** (119 mg, 0.258 mmol, 1 equiv.), HATU (196 mg, 0.516 mmol, 2 equiv.), DIPEA (134 µL, 0.774 mmol, 3 equiv.) and DMF (5 mL). The crude product was purified by column chromatography (SiO_2_, DCM/MeOD, 95/05) to afford the desired product as a yellow solid (159 mg, 76%).

**^1^H NMR (400 MHz, DMSO-*d*6)** *δ* 8.89 (t, *J* = 5.5 Hz, 1H), 8.53 (d, *J* = 8.5 Hz, 1H), 8.45 – 8.43 (m, 1H), 8.30 (t, *J* = 7.7 Hz, 2H), 8.25 (t, *J* = 8.6 Hz, 2H), 8.19 (dd, *J* = 8.0, 1.9 Hz, 2H), 8.15 (s, 1H), 8.08 (d, *J* = 7.7 Hz, 2H), 7.85 (t, *J* = 6.2 Hz, 1H), 7.80 (t, *J* = 8.0 Hz, 1H), 5.03 (d, *J* = 6.1 Hz, 2H), 4.40 – 4.29 (m, 1H), 3.73 (dd, *J* = 16.5, 5.9 Hz, 1H), 3.69 – 3.65 (m, 1H), 3.59 (dt, *J* = 13.1, 6.4 Hz, 2H), 3.46 (dd, *J* = 11.2, 2.3 Hz, 2H), 3.30 – 3.25 (m, 2H), 3.17 – 3.07 (m, 4H), 3.01 – 2.91 (m, 1H), 2.66 – 2.55 (m, 1H), 2.11 – 1.98 (m, 1H), 1.77 – 1.62 (m, 2H), 1.56 – 1.45 (m, 2H), 0.90 – 0.78 (m, 6H), NH unobserved.

**^13^C NMR (101 MHz, DMSO-*d*6)** *δ* 173.0, 172.2, 172.1, 168.9, 164.5, 160.9, 136.1, 134.4, 133.8, 131.3, 130.8, 130.4, 130.3, 129.8, 127.9, 127.4, 127.3, 126.8, 126.5, 126.2, 125.1, 125.0, 124.6, 124.6, 124.0, 124.0, 123.4, 120.7, 102.6, 79.2, 75.6, 64.2, 53.3, 51.9, 50.5, 42.1, 41.6, 31.3, 30.5, 28.4, 24.4, 23.6, 23.1, 22.1, 21.5.

**IR (cm^-1^)** 3264, 2956, 2934, 2859, 1710, 1685, 1531, 1448, 1371, 1299, , 1276, 1166, 1155, 1040, 955.

**HRMS** **(ESI-TOF) *m/z*** [M+H]^+^ Calcd for C_45_H_48_N_9_O_6_ 810.3727; Found 810.3723.

**Mp**. 147–149 °C.

**IV. Synthetic Procedure and analytical Data for cyclic compounds CyP2-13**

**General procedure E, for the intramolecular click-and-release reaction**

To a solution of sydnonimine derivative (1 equiv.) and triethanolamine (2 equiv.) in DMSO was added a solution of bathophenanthrolinedisulfonic acid disodium salt hydrate (0.4 equiv.) and CuSO_4_·5H_2_O (0.2 equiv.) in H_2_O. Then a solution of sodium ascorbate (3 equiv.) in H_2_O was added. ([C] = 0.036 M, DMSO/H_2_O, 1:1). The solution was stirred at the indicated time and temperature. The solvent was removed. To this mixture was added a solution of reduced graphene oxide in DCM (previously sonicated during 1 hour) (250 mg of rGO for 0.1 mmol of substrate). The dark solution was sonicated for 5 minutes and stirred overnight at room temperature. The solution was then filtrated and residue washed with additional DCM. The filtrate was then washed with water (5 times) and dried over MgSO_4_. The solvent was removed under reduced pressure to afford the pure product without further purification.

**Control experiments:**

1) Intermolecular reaction

Methyl (S)-3-(4-(4-((6-((tert-butoxycarbonyl)amino)-1-methoxy-1-oxohexan-2-yl)amino)-4-oxobutyl)-1H-pyrazol-1-yl)benzoatedione (P1)

C_27_H_38_N_4_O_7_

**MW**: 530.62 g.mol^-1^

**Yield**: 28%

Colorless oil

Following Procedure **E**, using **6** (141 mg, 0.296 mmol, 1 equiv.) **4** (105 mg, 0.296 mmol, 1 equiv.), triethanolamine (88 mg, 0.592 mmol, 2 equiv.), in DMSO (3 mL). Bathophenanthrolinedisulfonic acid disodium salt hydrate (63 mg, 0.118 mmol, 0.4 equiv.) and CuSO_4_·5H_2_O (14.7 mg, 0.059 mmol, 0.2 equiv.) in H_2_O (1.5 mL), and sodium ascorbate (176 mg, 0.888 mmol, 3 equiv.) in H_2_O (1.5 mL). (16 h, 37 °C). Reduced graphene oxyde (740 mg) in DCM (8 mL). The product was obtained as a colorles oil (44 mg, 28%).

**^1^H NMR (400 MHz, MeOD)** *δ* 8.31 (s, 1H), 8.11 (s, 1H), 7.95 – 7.87 (m, 2H), 7.60 (s, 1H), 7.55 (t, *J* = 7.9 Hz, 1H), 4.39 (dd, *J* = 9.0, 5.1 Hz, 1H), 3.92 (s, 3H), 3.70 (s, 3H), 3.05 – 2.98 (m, 2H), 2.59 (t, *J* = 8.1 Hz, 2H), 2.32 (t, *J* = 7.4 Hz, 2H), 1.99 – 1.89 (m, 2H), 1.86 – 1.76 (m, 1H), 1.74 – 1.61 (m, 1H), 1.51 – 1.29 (m, 4H), 1.39 (s, 9H), NH unobserved.

**^13^C NMR (101 MHz, MeOD)** *δ* 176.1, 174.2, 167.7, 158.5, 142.5, 141.6, 132.7, 130.8, 128.0, 127.3, 124.8, 124.1, 120.5, 79.9, 53.7, 52.9, 52.6, 40.9, 35.8, 32.0, 30.4, 28.7, 27.7, 24.3, 24.1.

**IR (cm^-1^)** 3278, 2956, 2868, 1709, 1685, 1450, 1300 , 1276, 1166, 1040, 958.

**HRMS** **(ESI-TOF) *m/z*** [M+H]^+^ Calcd for C_27_H_39_N_4_O_7_ 531.2819; Found 531.2821.

2) rGO work-up :

**Figure S1**. LC-MS analysis of the crude Cu-catalyzed cyclisation reaction of **S9** after 2 hours reaction, after rGO treatment and after H_2_O work-up and extraction.

(Z)-1^1^H-4,10-diaza-1(1,4)-pyrazola-2(1,3)-benzenacycloundecaphane-3,9-dione (CyP2)

C_16_H_18_N_4_O_2_

**MW**: 298.35 g.mol^-1^

**Yield**: 75%

White oil

Following Procedure **E**, using **S2** (85 mg, 0.142 mmol, 1 equiv.), triethanolamine (42 mg, 0.284 mmol, 2 equiv.), in DMSO (2 mL). Bathophenanthrolinedisulfonic acid disodium salt hydrate (30 mg, 0.057 mmol, 0.4 equiv.) and CuSO_4_·5H_2_O (7 mg, 0.028 mmol, 0.2 equiv.) in H_2_O (1 mL), and sodium ascorbate (84 mg, 0.426 mmol, 3 equiv.) in H_2_O (1 mL). (16 h, 37 °C). Reduced graphene oxyde (355 mg) in DCM (8 mL). The product was obtained as a white solid (32 mg, 75%).

**^1^H NMR (400 MHz, MeOD)** *δ* 7.90 – 7.85 (m, 2H), 7.69 – 7.60 (m, 3H), 7.53 (s, 1H), 4.29 (s, 2H), 3.21 – 3.14 (m, 2H), 2.36 – 2.25 (m, 2H), 2.08 – 1.91 (m, 2H), 1.65 – 1.50 (m, 2H), NH unobserved.

**^13^C NMR (101 MHz, MeOD)** *δ* 176.9, 172.7, 141.1, 140.6, 135.2, 132.1, 131.6, 128.1, 125.0, 123.2, 123.1, 46.1, 36.9, 35.4, 32.0, 25.5.

**IR (cm^-1^)** 3270, 2985, 2864, 1710, 1670, 1578, 1414, 1298, 1259, 1121, 1097, 1056, 985, 913.

**HRMS** **(ESI-TOF) *m/z*** [M+H]^+^ Calcd for C_16_H_19_N_4_O_2_ 299.1508; Found 299.1510.

(Z)-1^1^H-4,13-diaza-1(1,4)-pyrazola-2(1,3)-benzenacyclotetradecaphane-3,12-dione (CyP3)

C_19_H_24_N_4_O_2_

**MW**: 340.43 g.mol^-1^

**Yield**: 85%

Yellow oil

Following Procedure **E**, using **S3** (40 mg, 0.062 mmol, 1 equiv.), triethanolamine (18.5 mg, 0.124 mmol, 2 equiv.), in DMSO (1 mL). Bathophenanthrolinedisulfonic acid disodium salt hydrate (13.4 mg, 0.025 mmol, 0.4 equiv.) and CuSO_4_·5H_2_O (3.1 mg, 0.012 mmol, 0.2 equiv.) in H_2_O (0.5 mL), and sodium ascorbate (37 mg, 0.186 mmol, 3 equiv.) in H_2_O (0.5 mL). (16 h, 37 °C). Reduced graphene oxyde (155 mg) in DCM (5 mL). The product was obtained as a yellow solid (18 mg, 85%).

**^1^H NMR (400 MHz, CDCl_3_)** *δ* 7.91 (ddd, *J* = 8.1, 2.2, 1.1 Hz, 1H), 7.74 (d, *J* = 0.7 Hz, 1H), 7.64 (d, *J* = 7.6 Hz, 1H), 7.50 – 7.43 (m, 2H), 7.35 – 7.33 (m, 1H), 6.13 – 6.09 (m, 1H), 6.03 (t, *J* = 6.3 Hz, 1H), 4.33 (d, *J* = 6.4 Hz, 2H), 3.43 (dd, *J* = 11.1, 5.5 Hz, 2H), 2.21 (t, *J* = 7.2 Hz, 2H), 1.79 – 1.66 (m, 2H), 1.64 – 1.54 (m, 2H), 1.53 – 1.42 (m, 6H).

**^13^C NMR (101 MHz, MeOD)** *δ* 176.9, 170.8, 140.8, 140.7, 138.5, 131.5, 126.7, 126.4, 122.4, 119.5, 119.0, 39.9, 37.9, 35.1, 30.9, 30.0, 29.4, 28.3, 26.9.

**IR (cm^-1^)** 3270, 2986, 2864, 1710, 1680, 1414, 1370, 1298, 1121, 1099, 1057, 980.

**HRMS** **(ESI-TOF) *m/z*** [M+H]^+^ Calcd for C_19_H_25_N_4_O_2_ 341.1978; Found 341.1979.

(Z)-1^1^H-4,16-diaza-1(1,4)-pyrazola-2(1,3)-benzenacycloheptadecaphane-3,15-dione (CyP4)

C_22_H_30_N_4_O_2_

**MW**: 382.51 g.mol^-1^

**Yield**: 65%

Yellow oil

Following Procedure **E**, using **S4** (56 mg, 0.082 mmol, 1 equiv.), triethanolamine (24.4 mg, 0.164 mmol, 2 equiv.), in DMSO (1.2 mL). Bathophenanthrolinedisulfonic acid disodium salt hydrate (17.6 mg, 0.033 mmol, 0.4 equiv.) and CuSO_4_·5H_2_O (4.1 mg, 0.016 mmol, 0.2 equiv.) in H_2_O (0.6 mL), and sodium ascorbate (49 mg, 0.246 mmol, 3 equiv.) in H_2_O (0.6 mL). (16 h, 37 °C). Reduced graphene oxyde (205 mg) in DCM (7 mL). The product was obtained as a yellow oil (20 mg, 65%).

**^1^H NMR (400 MHz, CDCl_3_)** *δ* 7.99 (ddd, *J* = 8.1, 2.1, 1.1 Hz, 1H), 7.89 (s, 1H), 7.74 – 7.71 (m, 1H), 7.62 (s, 1H), 7.60 (t, *J* = 1.8 Hz, 1H), 7.54 (t, *J* = 8.0 Hz, 1H), 6.12 (t, *J* = 6.3 Hz, 1H), 5.86 (t, *J* = 5.9 Hz, 1H), 4.41 (d, *J* = 6.1 Hz, 2H), 3.54 (dd, *J* = 11.4, 5.9 Hz, 2H), 2.25 – 2.20 (m, 2H), 1.66 (dd, *J* = 26.6, 15.5 Hz, 4H), 1.39 – 1.19 (m, 12H).

**^13^C NMR (101 MHz, MeOD)** *δ* 176.1, 170.3, 142.0, 141.4, 138.2, 131.2, 127.5, 126.6, 123.8, 123.6, 118.2, 40.4, 36.9, 34.5, 31.1, 30.3, 29.9, 29.9, 29.7, 27.1, 26.8, 23.7.

**IR (cm^-1^)** 3238, 2986, 2958, 2866, 1708, 1686, 1515, 1429, 1370, 1300, 1100, 1057, 980.

**HRMS** **(ESI-TOF) *m/z*** [M+H]^+^ Calcd for C_22_H_31_N_4_O_2_ 383.2447; Found 383.2447.

(Z)-1^1^H-4,16,19-triaza-1(1,4)-pyrazola-2(1,3)-benzenacycloicosaphane-3,15,18-trione (CyP5)

C_24_H_33_N_5_O_3_

**MW**: 439.56 g.mol^-1^

**Yield**: 68%

Yellow oil

Following Procedure **E**, using **S5** (58 mg, 0.078 mmol, 1 equiv.), triethanolamine (23 mg, 0.156 mmol, 2 equiv.), in DMSO (1.1 mL). Bathophenanthrolinedisulfonic acid disodium salt hydrate (16.8 mg, 0.031 mmol, 0.4 equiv.) and CuSO_4_·5H_2_O (3.8 mg, 0.016 mmol, 0.2 equiv.) in H_2_O (0.6 mL), and sodium ascorbate (46 mg, 0.234 mmol, 3 equiv.) in H_2_O (0.6 mL). (16 h, 37 °C). Reduced graphene oxyde (195 mg) in DCM (10 mL). The product was obtained as a yellow oil (23 mg, 68%).

**^1^H NMR (400 MHz, MeOD)** *δ* 8.21 (s, 1H), 8.07 (t, *J* = 1.8 Hz, 1H), 7.95 (ddd, *J* = 8.1, 2.0, 0.9 Hz, 1H), 7.79 – 7.76 (m, 1H), 7.67 (s, 1H), 7.58 (t, *J* = 7.9 Hz, 1H), 4.36 (s, 2H), 3.83 (s, 2H), 3.44 (t, *J* = 6.3 Hz, 2H), 2.27 (t, *J* = 7.1 Hz, 2H), 1.70 – 1.54 (m, 4H), 1.48 – 1.40 (m, 2H), 1.38 – 1.26 (m, 10H), NH unobserved.

**^13^C NMR (101 MHz, MeOD)** *δ* 177.0, 175.3, 169.2, 142.3, 141.6, 132.5, 131.0, 128.0, 126.7, 123.3, 123.2, 118.2, 41.0, 36.5, 34.6, 30.0, 30.0, 29.7, 29.6, 29.4, 29.4, 29.2, 27.4, 26.1.

**IR (cm^-1^)** 3269, 2985, 2968, 2863, 1710, 1698, 1690, 1456, 1299, 1111, 1097, 1056, 986.

**HRMS** **(ESI-TOF) *m/z*** [M+H]^+^ Calcd for C_24_H_34_N_5_O_3_ 440.2662; Found 440.2665.

(Z)-1^1^H-4,16,22-triaza-1(1,4)-pyrazola-2(1,3)-benzenacyclotricosaphane-3,15,21-trione (CyP6)

C_27_H_39_N_5_O_3_

**MW**: 481.64 g.mol^-1^

**Yield**: 78%

Yellow oil

Following Procedure **E**, using **S6** (121 mg, 0.155 mmol, 1 equiv.), triethanolamine (46 mg, 0.310 mmol, 2 equiv.), in DMSO (2.2 mL). Bathophenanthrolinedisulfonic acid disodium salt hydrate (33 mg, 0.062 mmol, 0.4 equiv.) and CuSO_4_·5H_2_O (7.7 mg, 0.031 mmol, 0.2 equiv.) in H_2_O (1.1 mL), and sodium ascorbate (92 mg, 0.465 mmol, 3 equiv.) in H_2_O (1.1 mL). (16 h, 37 °C). Reduced graphene oxyde (387 mg) in DCM (10 mL). The product was obtained as a yellow oil (58 mg, 78%).

**^1^H NMR (400 MHz, MeOD/CD_2_Cl_2,_ 9:1)** *δ* 8.16 (s, 1H), 8.08 (s, 1H), 7.89 (d, *J* = 9.0 Hz, 1H), 7.76 (d, *J* = 7.8 Hz, 1H), 7.68 (s, 1H), 7.56 (t, *J* = 7.9 Hz, 1H), 4.31 (s, 2H), 3.43 (t, *J* = 6.2 Hz, 2H), 3.15 (t, *J* = 6.6 Hz, 2H), 2.25 (t, *J* = 7.2 Hz, 2H), 2.09 (t, *J* = 7.5 Hz, 2H), 1.71 – 1.57 (m, 4H), 1.57 – 1.43 (m, 4H), 1.40 – 1.14 (m, 12H), NH unobserved.

**^13^C NMR (101 MHz, MeOD/CD_2_Cl_2_ 9:1)** *δ* 176.2, 175.6, 169.0, 141.9, 141.1, 137.1, 130.8, 127.9, 126.3, 122.8, 122.8, 118.5, 40.7, 39.5, 38.8, 36.8, 36.1, 34.3, 29.6, 29.6, 29.5, 29.5, 29.5, 29.4, 27.2, 26.5, 23.8.

**IR (cm^-1^)** 3270, 2986, 2970, 2865, 1709, 1698, 1690, 1456, 1300, 1121, 1098, 1056, 983.

**HRMS** **(ESI-TOF) *m/z*** [M+H]^+^ Calcd for C_27_H_40_N_5_O_3_ 482.3232; Found 482.3134.

(E)-11H-7,10,13-trioxa-4-aza-1(1,4)-pyrazola-2(1,3)-benzenacyclotetradecaphan-3-one (CyP7)

C_17_H_21_N_3_O_4_

**MW**: 331.37 g.mol^-1^

**Yield**: 81%

Colorless oil

Following Procedure **E**, using **S7** (200 mg, 0.317 mmol, 1 equiv.), triethanolamine (94 mg, 0.634 mmol, 2 equiv.), in DMSO (5 mL). Bathophenanthrolinedisulfonic acid disodium salt hydrate (67 mg, 0.127 mmol, 0.4 equiv.) and CuSO_4_·5H_2_O (15.7 mg, 0.063 mmol, 0.2 equiv.) in H_2_O (2.5 mL), and sodium ascorbate (188 mg, 0.951 mmol, 3 equiv.) in H_2_O (2.5 mL). (16 h, 50 °C). Reduced graphene oxyde (792 mg) in DCM (10 mL). The product was obtained as a colorless oil (85 mg, 81%).

**^1^H NMR (400 MHz, MeOD)** *δ* 8.41 (d, *J* = 0.5 Hz, 1H), 8.01 (ddd, *J* = 8.0, 2.2, 1.0 Hz, 1H), 7.92 (t, *J* = 1.8 Hz, 1H), 7.74 – 7.69 (m, 1H), 7.61 (dd, *J* = 9.6, 6.1 Hz, 2H), 4.68 (s, 2H), 3.81 – 3.70 (m, 8H), 3.69 – 3.56 (m, 4H).

**^13^C NMR (101 MHz, MeOD)** *δ* 163.3, 140.7, 140.5, 137.7, 131.4, 127.6, 126.2, 123.9, 122.2, 119.7, 72.7, 72.5, 71.9, 70.9, 69.8, 65.0, 40.9.

**IR (cm^-1^)** 2985, 2859, 1710, 1416, 1345, 1298, 1220, 1121, 1097, 1056, 985.

**HRMS** **(ESI-TOF) *m/z*** [M+H]^+^ Calcd for C_17_H_22_N_3_O_4_ 332.1610; Found 332.1608.

**LC/MS control** showed very good purity of the product:

(Z)-11H-6,8-dioxa-4,10-diaza-1(1,4)-pyrazola-2,7(1,3),5,9(1,4)-tetrabenzenacyclotridecaphane-3,11-dione (CyP8)

C_31_H_24_N_4_O_4_

**MW**: 516.56 g.mol^-1^

**Yield**: 72%

Yellow sticky solid

Following Procedure **E**, using **S8** (63 mg, 0.078 mmol, 1 equiv.), triethanolamine (23 mg, 0.156 mmol, 2 equiv.), in DMSO (1.1 mL). Bathophenanthrolinedisulfonic acid disodium salt hydrate (16.8 mg, 0.031 mmol, 0.4 equiv.) and CuSO_4_·5H_2_O (3.8 mg, 0.016 mmol, 0.2 equiv.) in H_2_O (0.6 mL), and sodium ascorbate (46 mg, 0.234 mmol, 3 equiv.) in H_2_O (0.6 mL). (16 h, 37 °C). Reduced graphene oxyde (195 mg) in DCM (10 mL). The product was obtained as a yellow sticky solid (29 mg, 72%).

**^1^H NMR (400 MHz, CDCl_3_)** *δ* 7.98 (d, *J* = 6.7 Hz, 1H), 7.87 (s, 1H), 7.82 (s, 1H), 7.71 (d, *J* = 7.9 Hz, 1H), 7.56 – 7.45 (m, 4H), 7.37 (d, *J* = 9.0 Hz, 2H), 7.27 (t, *J* = 8.1 Hz, 1H), 7.07 (s, 1H), 6.96 (d, *J* = 8.9 Hz, 2H), 6.86 (d, *J* = 9.0 Hz, 2H), 6.78 – 6.73 (m, 1H), 5.99 (t, *J* = 2.3 Hz, 1H), 2.92 (t, *J* = 7.2 Hz, 2H), 2.60 (t, *J* = 7.2 Hz, 2H), NH unobserved.

**IR (cm^-1^)** 3270, 2986, 2856, 1710, 1691, 1514, 1470, 1349, 1300, 1258, 1209, 1089, 1056, 987.

**HRMS** **(ESI-TOF) *m/z*** [M+H]^+^ Calcd for C_31_H_25_N_4_O_4_ 517.1876; Found 517.1876.

**LC/MS control** showed very good purity of the product:

Methyl (9S,Z)-3,11-dioxo-11H-4,10-diaza-1(1,4)-pyrazola-2(1,3)-benzenacyclotetradecaphane-9-carboxylate (CyP9)

C_21_H_26_N_4_O_4_

**MW**: 398.46 g.mol^-1^

**Yield**: 76%

Colorless oil

Following Procedure **E**, using **S9** (120 mg, 0.172 mmol, 1 equiv.), triethanolamine (51 mg, 0.344 mmol, 2 equiv.), in DMSO (2.4 mL). Bathophenanthrolinedisulfonic acid disodium salt hydrate (36 mg, 0.068 mmol, 0.4 equiv.) and CuSO_4_·5H_2_O (8.4 mg, 0.034 mmol, 0.2 equiv.) in H_2_O (1.2 mL), and sodium ascorbate (102 mg, 0.516 mmol, 3 equiv.) in H_2_O (1.2 mL). (16 h, 37 °C). Reduced graphene oxyde (430 mg) in DCM (10 mL). The product was obtained as an colorless oil (52 mg, 76%).

**^1^H NMR (400 MHz, MeOD)** *δ* 8.20 (s, 1H), 8.04 – 7.93 (m, 2H), 7.88 (s, 1H), 7.68 (dd, *J* = 20.9, 5.7 Hz, 1H), 7.59 (s, 1H), 4.45 – 4.36 (m, 2H), 3.69 (s, *J* = 5.5 Hz, 3H), 3.44 – 3.34 (m, 2H), 2.78 – 2.53 (m, 2H), 2.43 – 2.28 (m, 2H), 2.26 – 2.14 (m, 2H), 1.82 – 1.72 (m, 2H), 1.70 – 1.59 (m, 2H), 1.52 – 1.38 (m, 1H), NH unobserved.

**^13^C NMR (101 MHz, MeOD)** *δ* 174.7, 173.7, 173.0, 141.3, 139.5, 136.8, 129.9, 126.5, 124.6, 122.5, 120.9, 118.7, 52.9, 51.2, 38.8, 33.9, 30.6, 28.6, 23.2, 21.1, 17.1.

**IR (cm^-1^)** 3260, 2986, 2970, 2865, 1710, 1690, 1680, 1469, 1378, 1299, 1121, 1099, 1056, 983, 960.

**HRMS** **(ESI-TOF) *m/z*** [M+H]^+^ Calcd for C_21_H_27_N_4_O_4_ 399.2032; Found 399.2032.

(5S,8S,Z)-5-isobutyl-8-methyl-1^1^H-4,7,10-triaza-1(1,4)-pyrazola-2(1,3)-benzenacycloundecaphane-3,6,9-trione (CyP10)

C_20_H_25_N_5_O_3_

**MW**: 383.45 g.mol^-1^

**Yield**: 68%

Colorless oil

Following Procedure **E**, using **S10** (60 mg, 0.088 mmol, 1 equiv.), triethanolamine (26 mg, 0.176 mmol, 2 equiv.), in DMSO (1.4 mL). Bathophenanthrolinedisulfonic acid disodium salt hydrate (18.8 mg, 0.035 mmol, 0.4 equiv.) and CuSO_4_·5H_2_O (4.4 mg, 0.017 mmol, 0.2 equiv.) in H_2_O (0.7 mL), and sodium ascorbate (52 mg, 0.264 mmol, 3 equiv.) in H_2_O (0.7 mL). (16 h, 50 °C). Reduced graphene oxyde (220 mg) in DCM (6 mL). The product was obtained as a colorless oil (22 mg, 68%).

**^1^H NMR (400 MHz, MeOD)** *δ* 8.08 (s, 1H), 8.03 (s, 1H), 7.75 (d, *J* = 7.7 Hz, 1H), 7.71 – 7.65 (m, 1H), 7.63 (s, 1H), 7.39 (t, *J* = 7.9 Hz, 1H), 4.44 (dd, *J* = 9.6, 5.2 Hz, 2H), 4.24 (q, *J* = 7.2 Hz, 2H), 1.88 – 1.76 (m, 2H), 1.74 – 1.66 (m, 1H), 1.44 (d, *J* = 6.7 Hz, 3H), 0.99 (dd, *J* = 13.9, 6.2 Hz, 6H), NH unobserved.

**^13^C NMR (101 MHz, MeOD)** *δ* 175.4, 174.5, 169.4, 141.4, 141.1, 135.6, 130.7, 127.8, 126.3, 122.9, 122.8, 118.3, 55.1, 51.3, 41.2, 30.7, 26.2, 23.3, 22.0, 16.8.

**IR (cm^-1^)** 3260, 2986, 2972, 2863, 1710, 1691, 1515, 1469, 1380, 1299, 1089, 1057, 986.

**HRMS** **(ESI-TOF) *m/z*** [M+H]^+^ Calcd for C_20_H_26_N_5_O_3_ 384.2036; Found 384.2036.

(5S,8S,Z)-5-benzyl-8-isobutyl-1^1^H-4,7,10-triaza-1(1,4)-pyrazola-2(1,3)-benzenacycloundecaphane-3,6,9-trione (CyP11)

C_26_H_29_N_5_O_3_

**MW**: 459.55 g.mol^-1^

**Yield**: 63%

Yellow oil

Following Procedure **E**, using **S11** (50 mg, 0.066 mmol, 1 equiv.), triethanolamine (19.6 mg, 0.132 mmol, 2 equiv.), in DMSO (1 mL). Bathophenanthrolinedisulfonic acid disodium salt hydrate (14 mg, 0.026 mmol, 0.4 equiv.) and CuSO_4_·5H_2_O (3.3 mg, 0.013 mmol, 0.2 equiv.) in H_2_O (0.5 mL), and sodium ascorbate (39 mg, 0.198 mmol, 3 equiv.) in H_2_O (0.5 mL). (16 h, 50 °C). Reduced graphene oxyde (200 mg) in DCM (6 mL). The product was obtained as a yellow solid (18.9 mg, 63%).

**^1^H NMR (400 MHz, MeOD)** *δ* 8.04 – 8.01 (m, 2H), 7.93 – 7.89 (m, 2H), 7.80 (s, 1H), 7.65 – 7.60 (m, 2H), 7.53 (s, 1H), 7.48 – 7.44 (m, 1H), 7.26 (t, *J* = 7.9 Hz, 2H), 4.55 (d, *J* = 15.3 Hz, 2H), 4.39 – 4.29 (m, 1H), 4.17 – 4.09 (m, 2H), 4.09 – 4.00 (m, 1H), 1.73 – 1.65 (m, 2H), 1.63 – 1.55 (m, 1H), 0.89 (dd, *J* = 11.8, 6.3 Hz, 6H), NH unobserved.

**^13^C NMR (101 MHz, MeOD)** *δ* 175.4, 174.8, 169.6, 141.5, 141.2, 130.7, 127.8, 127.8, 126.3, 125.0, 122.7, 122.3, 122.2, 119.4, 116.5, 113.6, 55.4, 51.1, 41.0, 34.8, 26.2, 23.3, 21.9, 16.7.

**IR (cm^-1^)** 3275, 2987, 2956, 2863, 1710, 1689, 1515, 1469, 1412, 1380, 1299, 1090, 986.

**HRMS** **(ESI-TOF) *m/z*** [M+H]^+^ Calcd for C_26_H_30_N_5_O_3_ 460.2349; Found 460.2353.

(5S,Z)-5-((1H-indol-3-yl)methyl)-11H-4,7,10-triaza-1(1,4)-pyrazola-2(1,3)-benzenacycloundecaphane-3,6,9-trione (CyP12)

C_24_H_22_N_6_O_3_

**MW**: 442.48 g.mol^-1^

**Yield**: 75%

Yellow solid

Following Procedure **E**, using **S12** (100 mg, 0.135 mmol, 1 equiv.), triethanolamine (40 mg, 0.270 mmol, 2 equiv.), in DMSO (2.1 mL). Bathophenanthrolinedisulfonic acid disodium salt hydrate (29 mg, 0.054 mmol, 0.4 equiv.) and CuSO_4_·5H_2_O (6.7 mg, 0.027 mmol, 0.2 equiv.) in H_2_O (1 mL), and sodium ascorbate (80 mg, 0.405 mmol, 3 equiv.) in H_2_O (1 mL). (16 h, 50 °C). Reduced graphene oxyde (337 mg) in DCM (6 mL). The product was obtained as a yellow solid (44 mg, 75%).

**^1^H NMR (400 MHz, MeOD)** *δ* 7.84 (s, 1H), 7.74 (t, *J* = 1.9 Hz, 1H), 7.68 (d, *J* = 7.8 Hz, 1H), 7.55 (s, 1H), 7.55 – 7.51 (m, 1H), 7.36 – 7.30 (m, 2H), 7.26 (s, 1H), 7.19 (t, *J* = 7.9 Hz, 1H), 7.12 – 7.07 (m, 1H), 7.01 (ddd, *J* = 8.0, 7.1, 1.0 Hz, 1H), 4.65 – 4.57 (m, 3H), 4.09 (dd, *J* = 21.3, 16.5 Hz, 2H), 3.54 – 3.41 (m, 2H), NH unobserved.

**^13^C NMR (101 MHz, MeOD)** *δ* 176.6, 172.1, 168.9, 140.6, 140.4, 138.1, 134.4, 130.5, 128.6, 127.0, 125.9, 124.8, 123.1, 122.6, 122.4, 119.9, 119.2, 117.4, 112.4, 110.6, 58.5, 44.4, 30.7, 24.2.

**IR (cm^-1^)** 3260, 2987, 2856, 1710, 1691, 1513, 1470, 1381, 1300, 1258, 1209, 1089, 1060, 986.

**HRMS** **(ESI-TOF) *m/z*** [M-H]^-^ Calcd for C_24_H_21_N_6_O_3_ 441.1675; Found 441.1677.

**LC/MS control** showed very good purity of the product:

(42S,7S,Z)-7-isobutyl-21H-6,9,12,15-tetraaza-2(1,4)-pyrazola-4(1,2)-pyrrolidina-1(1,3)-benzenacyclohexadecaphane-5,8,11,16-tetraone (CyP13)

C_26_H_35_N_7_O_4_

**MW**: 509.61 g.mol^-1^

**Yield**: 79%

Yellow solid

Following Procedure **E**, using **S13** (60 mg, 0.074 mmol, 1 equiv.), triethanolamine (22 mg, 0.148 mmol, 2 equiv.), in DMSO (1 mL). Bathophenanthrolinedisulfonic acid disodium salt hydrate (16 mg, 0.030 mmol, 0.4 equiv.) and CuSO_4_·5H_2_O (3.7 mg, 0.015 mmol, 0.2 equiv.) in H_2_O (0.5 mL), and sodium ascorbate (44 mg, 0.222 mmol, 3 equiv.) in H_2_O (0.5 mL). (16 h, 50 °C). Reduced graphene oxyde (185 mg) in DCM (6 mL). The product was obtained as an yellow solid (40 mg, 79%).

**^1^H NMR (400 MHz, CDCl_3_)** *δ* 8.60 (s, 1H), 8.14 – 8.10 (m, 2H), 8.08 (d, *J* = 9.4 Hz, 1H), 7.88 (d, *J* = 7.8 Hz, 1H), 7.59 (s, 1H), 7.57 – 7.52 (m, 1H), 7.47 (t, *J* = 5.4 Hz, 1H), 7.24 – 7.17 (m, 1H), 6.65 (t, *J* = 4.7 Hz, 1H), 4.69 – 4.59 (m, 1H), 3.97 (dd, *J* = 15.5, 4.8 Hz, 1H), 3.88 – 3.81 (m, 1H), 3.75 (d, *J* = 13.5 Hz, 1H), 3.68 (dd, *J* = 15.5, 6.5 Hz, 1H), 3.52 – 3.41 (m, 4H), 3.30 (dd, *J* = 10.4, 4.2 Hz, 1H), 3.17 (t, *J* = 7.3 Hz, 1H), 2.49 – 2.40 (m, 1H), 2.30 – 2.19 (m, 1H), 1.87 – 1.78 (m, 2H), 1.71 – 1.59 (m, 2H), 1.58 – 1.49 (m, 2H), 0.90 (dd, *J* = 11.9, 6.2 Hz, 6H).

**^13^C NMR (101 MHz, CDCl_3_)** *δ* 175.0, 174.5, 169.7, 166.9, 141.1, 140.0, 135.3, 130.4, 127.7, 126.1, 122.4, 121.8, 115.0, 67.4, 54.0, 50.8, 49.3, 44.6, 42.4, 39.7, 38.8, 31.0, 25.0, 24.8, 23.0, 22.2.

**IR (cm^-1^)** 3269, 2979, 2954, 2836, 1711, 1689, 1654, 1515, 1456, 1310, 1296, 1040, 944.

**HRMS** **(ESI-TOF) *m/z*** [M+H]^+^ Calcd for C_26_H_36_N_7_O_4_ 510.2829; Found 510.2831.

**Mp**. 129–131 °C.

**V. NMR Spectra**

^1^H NMR spectrum of compound **2** (solvent CDCl_3_, 400 MHz)

^13^C NMR spectrum of compound **2** (solvent CDCl_3_, 100 MHz)

^1^H NMR spectrum of compound **3** (solvent DMSO-*d*6, 400 MHz)

^13^C NMR spectrum of compound **3** (solvent DMSO-*d*6, 100 MHz)

^1^H NMR spectrum of compound **4** (solvent CDCl_3_, 400 MHz)

^13^C NMR spectrum of compound **4** (solvent CDCl_3_, 100 MHz)

^1^H NMR spectrum of compound **5** (solvent DMSO-*d*6, 400 MHz)

^1^H NMR spectrum of compound **6** (solvent MeOD, 400 MHz)

^13^C NMR spectrum of compound **6** (solvent MeOD, 100 MHz)

^1^H NMR spectrum of compound **10** (solvent MeOD, 400 MHz)

^13^C NMR spectrum of compound **10** (solvent MeOD, 100 MHz)

^1^H NMR spectrum of compound **11** (solvent MeOD, 400 MHz)

^13^C NMR spectrum of compound **11** (solvent MeOD, 100 MHz)

^1^H NMR spectrum of compound **12** (solvent MeOD, 400 MHz)

^13^C NMR spectrum of compound **12** (solvent MeOD, 100 MHz)

^1^H NMR spectrum of compound **13** (solvent MeOD, 400 MHz)

^13^C NMR spectrum of compound **13** (solvent MeOD, 100 MHz)

^1^H NMR spectrum of compound **14** (solvent MeOD, 400 MHz)

^13^C NMR spectrum of compound **14** (solvent MeOD, 100 MHz)

^1^H NMR spectrum of compound **15** (solvent MeOD, 400 MHz)

^13^C NMR spectrum of compound **15** (solvent MeOD, 100 MHz)

^1^H NMR spectrum of compound **16** (solvent CDCl_3_, 400 MHz)

^13^C NMR spectrum of compound **16** (solvent CDCl_3_, 100 MHz)

^1^H NMR spectrum of compound **18** (solvent MeOD, 400 MHz)

^13^C NMR spectrum of compound **18** (solvent MeOD, 100 MHz)

^1^H NMR spectrum of compound **19** (solvent MeOD, 400 MHz)

^13^C NMR spectrum of compound **19** (solvent MeOD, 100 MHz)

^1^H NMR spectrum of compound **20** (solvent MeOD, 400 MHz)

^13^C NMR spectrum of compound **20** (solvent MeOD, 100 MHz)

^1^H NMR spectrum of compound **21** (solvent MeOD, 400 MHz)

^13^C NMR spectrum of compound **21** (solvent MeOD, 100 MHz)

^1^H NMR spectrum of compound **22** (solvent MeOD, 400 MHz)

^13^C NMR spectrum of compound **22** (solvent MeOD, 100 MHz)

^1^H NMR spectrum of compound **23** (solvent MeOD, 400 MHz)

^13^C NMR spectrum of compound **23** (solvent MeOD, 100 MHz)

^1^H NMR spectrum of compound **24** (solvent MeOD, 400 MHz)

^13^C NMR spectrum of compound **24** (solvent MeOD, 100 MHz)

^1^H NMR spectrum of compound **25** (solvent MeOD, 400 MHz)

^13^C NMR spectrum of compound **25** (solvent MeOD, 100 MHz)

^1^H NMR spectrum of compound **26** (solvent MeOD, 400 MHz)

^13^C NMR spectrum of compound **26** (solvent MeOD, 100 MHz)

^1^H NMR spectrum of compound **27** (solvent MeOD, 400 MHz)

^13^C NMR spectrum of compound **27** (solvent MeOD, 100 MHz)

^1^H NMR spectrum of compound **28** (solvent MeOD, 400 MHz)

^13^C NMR spectrum of compound **28** (solvent MeOD, 100 MHz)

^1^H NMR spectrum of compound **29** (solvent MeOD, 400 MHz)

^13^C NMR spectrum of compound **29** (solvent MeOD, 100 MHz)

^1^H NMR spectrum of compound **30** (solvent MeOD, 400 MHz)

^13^C NMR spectrum of compound **30** (solvent MeOD, 100 MHz)

^1^H NMR spectrum of compound **32** (solvent MeOD, 400 MHz)

^13^C NMR spectrum of compound **32** (solvent MeOD, 100 MHz)

^1^H NMR spectrum of compound **33** (solvent MeOD, 400 MHz)

^13^C NMR spectrum of compound **33** (solvent MeOD, 100 MHz)

^1^H NMR spectrum of compound **34** (solvent MeOD, 400 MHz)

^13^C NMR spectrum of compound **34** (solvent MeOD, 100 MHz)

^1^H NMR spectrum of compound **35** (solvent MeOD, 400 MHz)

^13^C NMR spectrum of compound **35** (solvent MeOD, 100 MHz)

^1^H NMR spectrum of compound **36** (solvent MeOD, 400 MHz)

^13^C NMR spectrum of compound **36** (solvent MeOD, 100 MHz)

^1^H NMR spectrum of compound **S1** (solvent DMSO-*d*6, 400 MHz)

^13^C NMR spectrum of compound **S1** (solvent DMSO-*d*6, 100 MHz)

^1^H NMR spectrum of compound **S2** (solvent DMSO-*d*6, 400 MHz)

^13^C NMR spectrum of compound **S2** (solvent DMSO-*d*6, 100 MHz)

^1^H NMR spectrum of compound **S3** (solvent DMSO-*d*6, 400 MHz)

^13^C NMR spectrum of compound **S3** (solvent DMSO-*d*6, 100 MHz)

^1^H NMR spectrum of compound **S4** (solvent DMSO-*d*6, 400 MHz)

^13^C NMR spectrum of compound **S4** (solvent DMSO-*d*6, 100 MHz)

^1^H NMR spectrum of compound **S5** (solvent DMSO-*d*6, 400 MHz)

^13^C NMR spectrum of compound **S5** (solvent DMSO-*d*6, 100 MHz)

^1^H NMR spectrum of compound **S6** (solvent DMSO-*d*6, 400 MHz)

^13^C NMR spectrum of compound **S6** (solvent DMSO-*d*6, 100 MHz)

^1^H NMR spectrum of compound **S7** (solvent DMSO-*d*6, 400 MHz)

^13^C NMR spectrum of compound **S7** (solvent DMSO-*d*6, 100 MHz)

^1^H NMR spectrum of compound **S8** (solvent DMSO-*d*6, 400 MHz)

grease

^13^C NMR spectrum of compound **S8** (solvent DMSO-*d*6, 100 MHz)

^1^H NMR spectrum of compound **S9** (solvent CDCl_3_, 400 MHz)

^13^C NMR spectrum of compound **S9** (solvent CDCl_3_, 100 MHz)

^1^H NMR spectrum of compound **S10** (solvent CDCl_3_, 400 MHz)

^13^C NMR spectrum of compound **S10** (solvent CDCl_3_/MeOD (1:1), 100 MHz)

^1^H NMR spectrum of compound **S11** (solvent DMSO-*d*6, 400 MHz)

^13^C NMR spectrum of compound **S11** (solvent DMSO-*d*6, 100 MHz)

^1^H NMR spectrum of compound **S12** (solvent DMSO-*d*6, 400 MHz)

^13^C NMR spectrum of compound **S12** (solvent DMSO-*d*6, 100 MHz)

^1^H NMR spectrum of compound **S13** (solvent DMSO-*d*6, 400 MHz)

grease

^13^C NMR spectrum of compound **S13** (solvent DMSO-*d*6, 100 MHz)

^1^H NMR spectrum of compound **P1** (solvent MeOD, 400 MHz)

^13^C NMR spectrum of compound **P1** (solvent MeOD, 100 MHz)

^1^H NMR spectrum of compound **CyP2** (solvent MeOD, 400 MHz)

^13^C NMR spectrum of compound **CyP2** (solvent MeOD, 100 MHz)

^1^H NMR spectrum of compound **CyP3** (solvent CDCl_3_, 400 MHz)

^13^C NMR spectrum of compound **CyP3** (solvent MeOD, 100 MHz)

^1^H NMR spectrum of compound **CyP4** (solvent CDCl_3_, 400 MHz)

^13^C NMR spectrum of compound **CyP4** (solvent MeOD, 100 MHz)

^1^H NMR spectrum of compound **CyP5** (solvent MeOD, 400 MHz)

^13^C NMR spectrum of compound **CyP5** (solvent MeOD, 100 MHz)

^1^H NMR spectrum of compound **CyP6** (solvent MeOD/CD_2_Cl_2_ (9:1), 100 MHz)

^13^C NMR spectrum of compound **CyP6** (solvent MeOD/CD_2_Cl_2_ (9:1), 100 MHz)

^1^H NMR spectrum of compound **CyP7** (solvent MeOD, 400 MHz)

^13^C NMR spectrum of compound **CyP7** (solvent MeOD, 100 MHz)

^1^H NMR spectrum of compound **CyP8** (solvent CDCl_3_, 400 MHz)

^13^C NMR spectrum of compound **CyP8** (solvent CDCl3, 100 MHz)

^1^H NMR spectrum of compound **CyP9** (solvent MeOD, 400 MHz)

^13^C NMR spectrum of compound **CyP9** (solvent MeOD, 100 MHz)

^1^H NMR spectrum of compound **CyP10** (solvent MeOD, 400 MHz)

^13^C NMR spectrum of compound **CyP10** (solvent MeOD, 100 MHz)

^1^H NMR spectrum of compound **CyP11** (solvent MeOD, 400 MHz)

^13^C NMR spectrum of compound **CyP11** (solvent MeOD, 100 MHz)

^1^H NMR spectrum of compound **CyP12** (solvent MeOD, 400 MHz)

^13^C NMR spectrum of compound **CyP12** (solvent MeOD, 100 MHz)

^1^H NMR spectrum of compound **CyP13** (solvent MeOD, 400 MHz)

^13^C NMR spectrum of compound **CyP13** (solvent MeOD, 100 MHz)

**VI. Crystal structures of compounds CyP2, 3 and 7**

Data collections were performed at 100(2) K on a Bruker D8 Quest diffractometer using an Incoatec Microfocus Source (IμS 3.0 Mo) and a PHOTON III area detector, and operated with APEX4.^[[3]](#footnote-3)^ The data were processed with SAINT,^[[4]](#footnote-4)^ and empirical absorption corrections were made with SADABS.^[[5]](#footnote-5),^^[[6]](#footnote-6)^ The structures were solved by intrinsic phasing with SHELXT,^[[7]](#footnote-7)^ and refined by full-matrix least-squares on *F*^2^ with SHELXL,^[[8]](#footnote-8)^ using the ShelXle interface.^[[9]](#footnote-9)^ The hydrogen atoms bound to oxygen and nitrogen atoms were retrieved from residual electron density maps and they were refined either freely or with geometric restraints. All other hydrogen atoms in all compounds were introduced at calculated positions and treated as riding atoms with an isotropic displacement parameter equal to 1.2 times that of the parent atom (1.5 for CH_3_).

**Crystal Structure Report for compound CyP2**


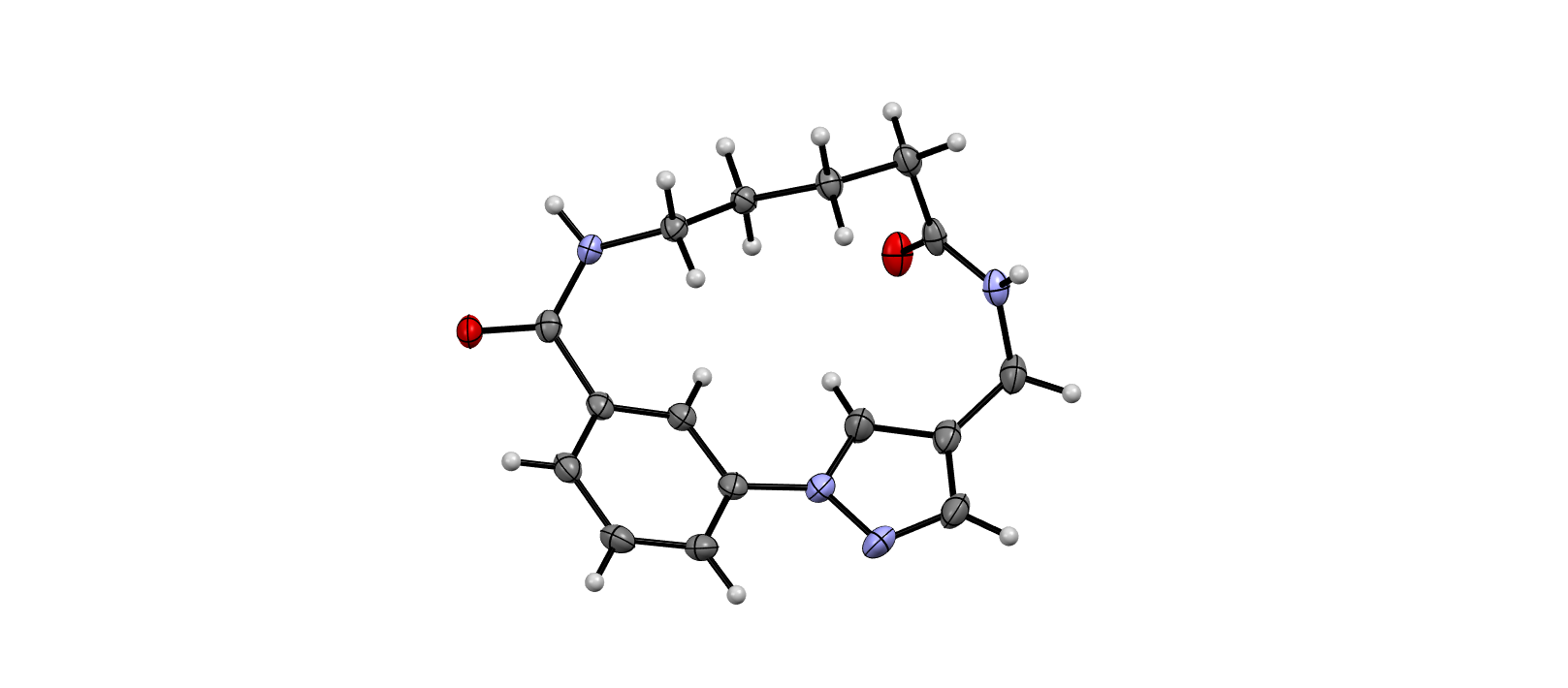


**Crystal Structure Report for compound CyP3**

**
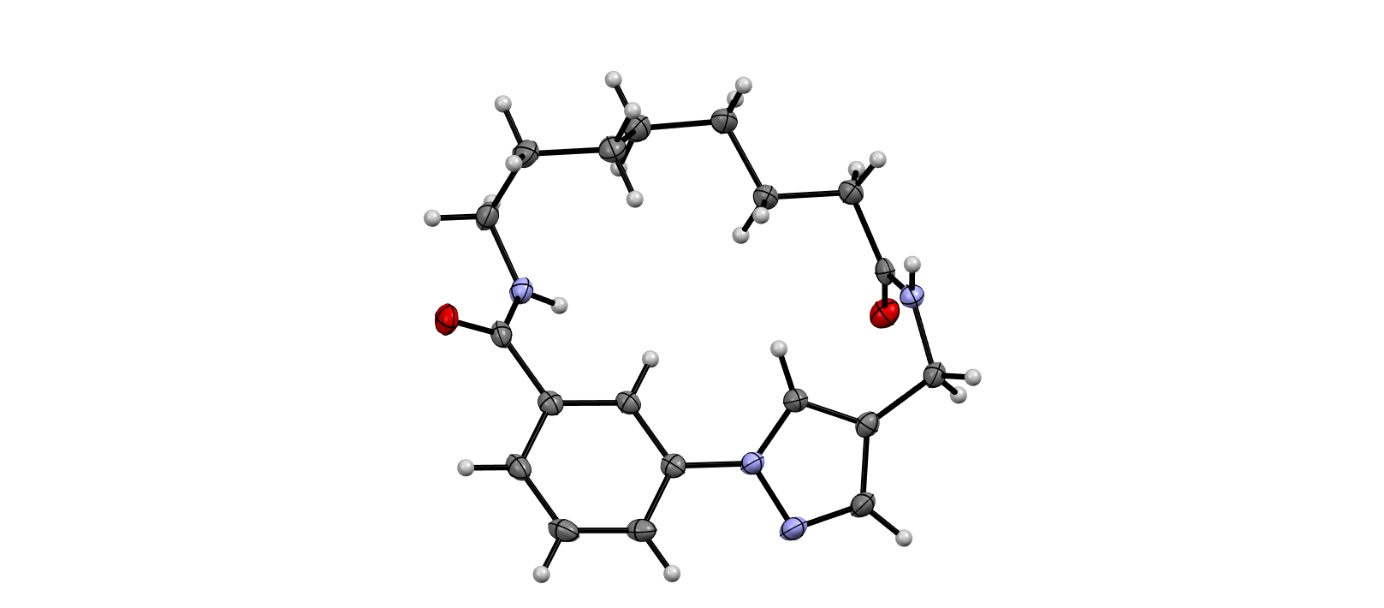
**

**Crystal Structure Report for compound CyP7**

**
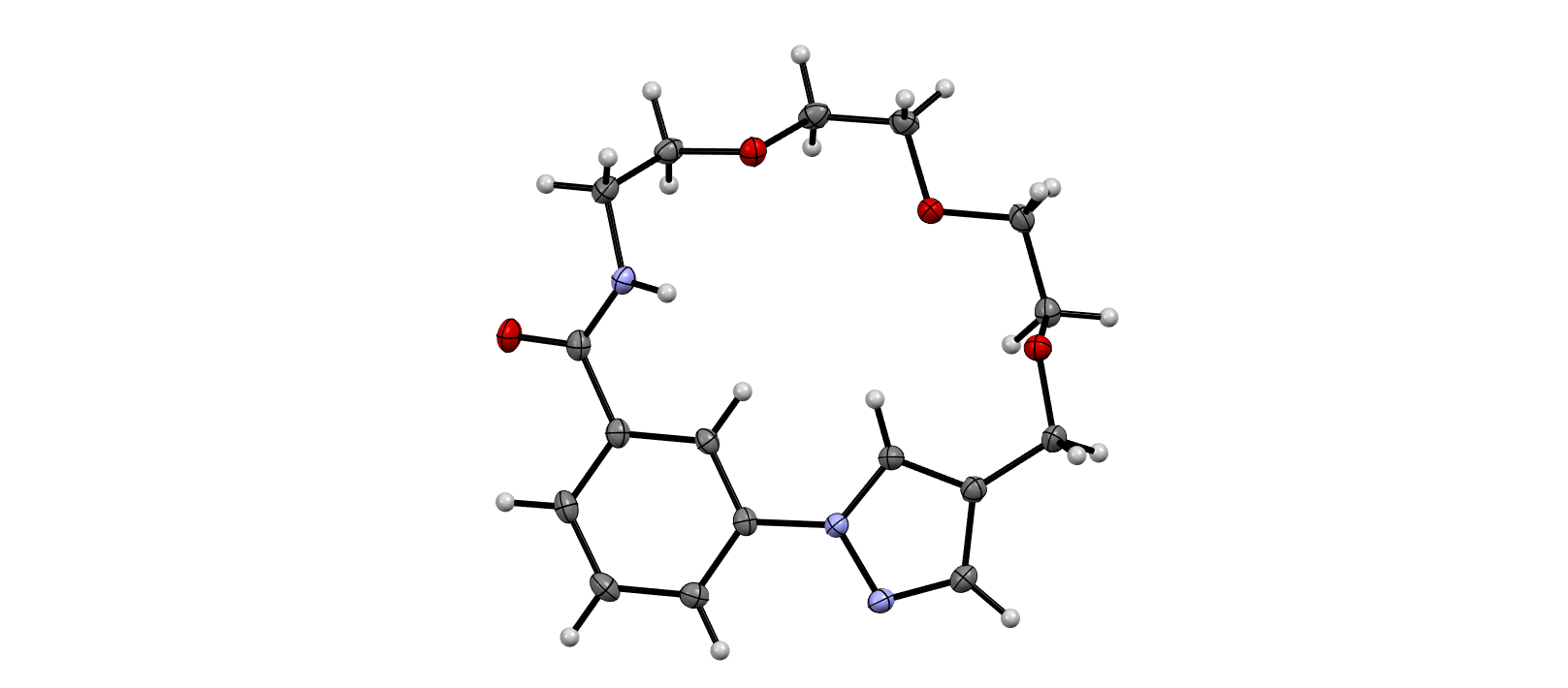
**

1. F. J. Dekker, M. Ghizzoni, N. van der Meer, R. Wisastra, H. J. Haisma, *Bioorg. Med. Chem.* **2009**, *17*, 460–466. [↑](#footnote-ref-1)
2. *Chinese Chemical Letters.* **2009**, *20*, 1345–1347. [↑](#footnote-ref-2)
3. *SAINT*, *ver. 8.40A*, Bruker Nano, Madison, WI, 2019. [↑](#footnote-ref-3)
4. *SADABS*, *ver. 2016/2*, Bruker AXS, Madison, WI, 2016. [↑](#footnote-ref-4)
5. L. Krause, R. Herbst-Irmer, G. M. Sheldrick and D. Stalke, *J. Appl. Crystallogr.*, 2015, **48**, 3. [↑](#footnote-ref-5)
6. G. M. Sheldrick, *Acta Crystallogr., Sect. A*, 2015, **71**, 3. [↑](#footnote-ref-6)
7. G. M. Sheldrick, *Acta Crystallogr., Sect. C*, 2015, **71**, 3. [↑](#footnote-ref-7)
8. C. B. Hübschle, G. M. Sheldrick and B. Dittrich, *J. Appl. Crystallogr.*, 2011, **44**, 1281. [↑](#footnote-ref-8)
9. C. B. Hübschle, G. M. Sheldrick and B. Dittrich, *J. Appl. Crystallogr.*, 2011, **44**, 1281 [↑](#footnote-ref-9)
